# Characterising PMP22-Proximal Partners in a Schwann Cell Model of Charcot–Marie–Tooth Disease Type1A

**DOI:** 10.3390/biology14111552

**Published:** 2025-11-05

**Authors:** Ian Holt, Nicholas Emery, Monte A. Gates, Sharon J. Brown, Sally L. Shirran, Heidi R. Fuller

**Affiliations:** 1 Wolfson Centre for Inherited Neuromuscular Disease, TORCH Centre, RJAH Orthopaedic Hospital, Oswestry SY10 7AG, UK; ian.holt@nhs.net (I.H.); nicholas.emery1@nhs.net (N.E.); s.j.owen@keele.ac.uk (S.J.B.); 2 School of Medicine, Keele University, Keele ST5 5BG, UK; m.a.gates@keele.ac.uk; 3 School of Life Sciences, Keele University, Keele ST5 5BG, UK; 4 BSRC Mass Spectrometry and Proteomics Facility, University of St Andrews, St Andrews KY16 9ST, UK; ss101@st-andrews.ac.uk; 5 School of Allied Health Professions and Pharmacy, Keele University, Keele ST5 5BG, UK

**Keywords:** Charcot-Marie-Tooth disease 1A, PMP22, ITGA2, ITGA7, Schwann cells, BioID2, proteomics

## Abstract

**Simple Summary:**

Charcot–Marie–Tooth disease (CMT) encompasses a group of progressive and variable genetic diseases in which the peripheral nerves controlling movement and sensation in the legs and arms become damaged. Symptoms include muscle weakness and sensation loss in the feet and legs and sometimes in the hands, which can be detrimental to patient mobility. Schwann cells form insulating sheaths which protect nerve fibres that carry electrical impulses. The most common form of CMT is type 1A (CMT1A), caused by the overproduction of the PMP22 protein by Schwann cells, leading to nerve insulation loss and damage to peripheral nerves. Current models of CMT1A have limitations including high cost, variability and being time-consuming to perform. To address this, we developed a stable Schwann cell line which overexpresses the PMP22 protein. Using this cell model, we identified proteins which interact with, or are in very close proximity to, the overexpressed PMP22 protein. Some of these proteins are found on the surface of Schwann cells, which anchor the cells to the surrounding supportive material and are important for the maintenance of the insulating sheath. This cell model may complement existing models and be used to study the mechanisms of action of the disease and potentially to identify therapies for CMT1A.

**Abstract:**

Charcot–Marie–Tooth disease type 1A (CMT1A) is a hereditary condition caused by the duplication of the *PMP22* gene. Overexpression of peripheral myelin protein 22 in Schwann cells leads to myelin sheath defects and axonal loss. We have produced a cell model to facilitate studies of the molecular mechanisms involved in PMP22 accumulation and clearance. Our model is a stably transfected, clonal, immortalised human Schwann cell line with overexpressed levels of PMP22 fusion protein. A control-transfected cell line (vector lacking *PMP22*) was also produced. *PMP22*-transfected cells had reduced levels of mitosis, with the PMP22 fusion protein concentrated in punctate aggregates in the cytoplasm and expressed at the plasma membranes, which were often irregular and spindly. In contrast, control cells (control-transfected and parent cell lines) generally had smooth and regular plasma membrane morphology. Culturing in the presence of NRG1 and forskolin lead to upregulation of markers of myelination potential in the control cells. These markers were more variable in the cells stably transfected with *PMP22*, including decreased levels of transcripts of *SOX10*, *JUN*, *S100B* and *NGFR*, but increased levels of *MPZ* and *EGR2* compared to controls. Using proximity-dependent biotin identification (BioID2), several hundred proteins were identified in the proximity of the overexpressed PMP22, of which 291 significant proteins were only detected in the proximity of PMP22 and not in that of control pull-downs. Among the most significantly enriched PMP22-interacting proteins were integrins alpha-2 (ITGA2) and alpha-7 (ITGA7), which play a role in myelination via their interactions with the extracellular matrix. The presence of ITGA2 in just the *PMP22*-transfected fraction was confirmed by western blot. Some of the proteins were associated with several enriched molecular pathways, including molecular transport and protein trafficking, and may represent potential therapeutic targets for CMT1A by promoting the degradation and enhanced trafficking of PMP22.

## 1. Introduction

Charcot–Marie–Tooth disease (CMT) is a heterogeneous group of peripheral neuropathies with a prevalence of approximately 1 in 2500 [1]. There is significant variability in the severity of symptoms, but pathological features may include slow progressive muscle weakness and wasting in the anterior legs and feet, high arched feet (pes cavus), hammertoes and difficulty walking or running. As the disease progresses, weakness may move up the legs and affect the hands. Symptoms may also include numbness, decreased reflexes and vibratory sensory loss [2]. The major types of CMT disease are classified as type 1 (CMT1; ~57% of cases), which is primarily associated with demyelination, or type 2 (CMT2; ~27% of cases), which is associated with axonal degeneration. CMT type 1A (CMT1A), the most common form of CMT, accounting for ~70% of CMT1 and around 40% of all CMT cases, is caused by the duplication of the *PMP22* gene. This autosomal dominant, gain-of-function disease typically results in three copies of the *PMP22* gene, and overexpression of peripheral myelin protein 22 (PMP22) in Schwann cells, which is associated with demyelination [2]. The myelin sheath is formed from Schwann cell plasma membranes that are greatly extended and modified and wrap around nerve axons in a spiral manner. PMP22 is a tetraspan integral membrane protein which is dynamically controlled and highly expressed in the plasma membrane of myelinating Schwann cells [3,4]. Glycosylated PMP22 is transported to the plasma membrane and has a role in the development and maintenance of myelin [4,5].

Overexpression of PMP22 via “gene dosage” is the widely proposed mechanism of myelin sheath defects and subsequent axonal loss and muscle atrophy in CMT1A, supported by findings of increased PMP22 protein [6,7] and messenger RNA in CMT1A patients in sural nerve biopsies [8]. Transgenic mouse studies have shown a positive correlation between the expression of PMP22 and the severity of neuropathy [9]. PMP22 is prone to misfolding, and only 20% of newly expressed protein traffics correctly to the cell surface [10]. The precise roles are yet to be elucidated, but one function of PMP22 is thought to be as a structural component of compact myelin [11]. Overabundance of PMP22 results in an accumulation of misfolded protein in the endoplasmic reticulum (ER) due to oversaturation of the ER quality control network, leading to insufficient trafficking or degradation, resulting in cell stress and toxicity [10]. In addition to the overexpression of PMP22 causing CMT1A, dominant missense mutations in the *PMP22* gene may cause CMT1E (<5% of CMT1 cases), and the loss of function in the *PMP22* gene causes hereditary neuropathy with pressure palsies (HNPP) [5]. The reduction in PMP22 protein expression is a promising approach for potential CMT1A therapy, as demonstrated by the shRNA knockdown of *Pmp22* in a rodent model of CMT1A, which resulted in increased myelination and the prevention of motor and sensory impairment [12]. Potential therapies need to be controlled to ensure that detrimental underexpression of PMP22 does not occur. However, therapy development is hindered by limited insights into the molecular pathways involved in PMP22 accumulation and clearance, and the limitations of current disease models for high-throughput phenotypic-based drug screening studies.

Current models of CMT1A tend to involve the use of either transgenic rodent models overexpressing PMP22 protein, subcultures from the rodent models, or CMT1A patient-derived human induced pluripotent stem cells (hiPSCs). Transgenic rats that overexpress PMP22 in Schwann cells have high levels of PMP22, both intracellularly and at the Schwann cell membrane. These animals show gate abnormalities, Schwann cell hypertrophy, reduced nerve conduction and muscle weakness [13,14]. Similar abnormalities were also seen in transgenic mouse models that overexpress human PMP22 [15] and mouse PMP22 [16,17]. Additionally, the Trembler J mouse model, which carries an autosomal dominant point mutation in *Pmp22*, results in a severe model of CMT1E [18]. Despite preclinical achievements, rodent models are not well suited to drug screening studies due to the high time requirements, costs and potential variability [19]. In vitro models have also been developed, including stable CMT1A patient-derived hiPSCs derived from fibroblasts [20,21,22] and from peripheral blood mononuclear cells [23]. Non-stable models of CMT1A include monolayer co-cultures of rat dorsal root ganglion sensory neurons and Schwann cells from CMT1A transgenic rats [24,25], dorsal root ganglion explant cultures from *Pmp22* transgenic mice [26,27], and rat Schwann cells [28] and HeLa cells [29] transiently transfected with *PMP22*-containing vectors. While these models have each contributed valuable molecular insights, the variability that occurs with hiPSC and non-stable cell models, and the limitation of using immature cells as a model for a late-onset disease, highlight the need for additional, complementary in vitro disease models [19]. To address this, we have established a stably transfected, clonal, immortalised human Schwann cell line with overexpressed levels of PMP22 fusion protein. We present our findings from the proteomic characterisation of overexpressed PMP22-proximal partners.

## 2. Materials and Methods

### 2.1. Immortalised Schwann Cell Culture and Geneticin “Kill Curve” Determination

An immortalised human Schwann cell line (hTERT ipn02.3 2λ; ATCC CRL-3392), from the sural nerve of a female donor with non-malignant plexiform neurofibroma, was obtained from the American Type Culture Collection (ATCC) (LGC Standards, Middlesex, UK). The immortalised Schwann cells were cultured in DMEM (Cat No: 31966-021; Gibco, Thermo Fisher Scientific, Paisley, UK) with 10% foetal bovine serum (Cat No: 10270; Gibco), 100 U/mL Penicillin, 100 µg/mL Streptomycin (Cat No: 15140-122; Gibco) and Minimum Essential Medium Non-Essential Amino Acids (Cat No: 11140-035; Gibco) at 37 °C in a humidified incubator at 5% CO_2_. For the “kill curve” determination, immortalised Schwann cells were cultured for 1 day in 24-well plates to around 70% confluency and then the medium was changed to contain a range of concentrations (0 to 1000 µg/mL) of the selective antibiotic geneticin (G418 sulfate; Cat No: 10131-027; Gibco). Cells were examined daily and the culture medium was removed and replaced with fresh medium with geneticin every 3 or 4 days. It was found that 300 µg/mL was the lowest concentration of geneticin that killed all the cells within 7 to 10 days.

### 2.2. Expression Vectors

PMP22 and control mammalian expression plasmids (pRP[Exp]) were manufactured by VectorBuilder Inc. (Chicago, IL, USA) (Figure S1). The vectors included a CMV promoter, neomycin resistance gene for eukaryotic selection and ampicillin resistance gene for prokaryotic selection. The PMP22 expression vector (VB210906-1101thx) contained an inserted open reading frame consisting of 3 components: enhanced green fluorescent protein (EGFP; NCBI Accession No: AFA52654.1), Homo Sapiens peripheral myelin protein 22 (hPMP22; Accession No: NM_153322.3) and BioID2 (Accession No: MQ048429.1), with flexible linkers (3 tandem GGGGS) separating each of the 3 components. The control expression vector (VB210906-1105mys) contained an open reading frame with just EGFP and BioID2, separated by a flexible linker. Plasmids were prepared by standard techniques (Endofree plasmid maxi; Cat No: 12362; Qiagen Ltd., Manchester, UK) to yield expected sizes of PMP22 and control plasmids of 6614 bp and 6086 bp, respectively.

### 2.3. Transfection of Schwann Cells and Establishment of Stable Cell Lines

Immortalised Schwann cells were transfected with PMP22 or control plasmids using the MIR 51000 Ingenio EZporator Electroporation System (Geneflow Ltd., Lichfield, Staffordshire, UK) following manufacturer’s instructions. Following electroporation, cells were immediately added to the culture medium at a final density of 2 × 10^4^ cells/mL and 0.5 mL cells/well added to 24-well plates. Cells were examined daily. After 2 days the cells were 20 to 50% confluent, at which point the medium was removed from the wells and replaced with fresh medium containing 300 µg/mL geneticin (Day 0). Geneticin should kill cells not expressing neomycin resistance. The medium was removed and replaced with fresh medium every 3 or 4 days. After 4 days in the presence of geneticin, there was non-adherent cell debris, and viable cell confluency was between 60 and 90%. Cell culture was continued for 8 weeks in the presence of geneticin to select for stable transfectants. Cells were examined with a JuLI smart fluorescent cell analyser (Digital Bio, Watertown, MA, USA) to identify wells containing cells expressing EGFP. Wells containing EGFP-expressing cells were cloned by limiting dilution. Cells were diluted to approximately 1 cell per well in 96-well plates and were fed with additional medium after 1 week. As an indication that the cell dilutions were correct, 40 to 60% of wells did not contain any cells. After the first cloning, cells were cultured for a total of 2 weeks prior to EGFP-expressing cells being cloned for a second time. The initial screen was EGFP expression, followed by a second screen of immunofluorescence staining for the BioID2 tag. When all cells in a well screened positive, they were considered to be clonal. Cloning twice was sufficient to obtain clonal populations of the PMP22 and control stable transfectants. Clonal populations were then expanded for storage and for maintenance in cell culture. For cell counting, equal volumes of cell suspension and 0.4% trypan blue were mixed and added to a hemacytometer to determine the number of unstained, viable cells. Cell proliferation data was analysed by two-way ANOVA using GraphPad Prism (v9.5.1; GraphPad Software, San Diego, CA, USA), with cell line as the between-subject factor and time in culture as the within-subject factor. When significant main or interaction effects were identified, Tukey’s multiple comparisons post hoc test was applied. Statistical significance was set at *p* < 0.05.

### 2.4. Immunofluorescence Microscopy

Cells were seeded onto glass coverslips at an initial density of 2 × 10^4^ cells/mL. At the end of the culture period, adherent cells on coverslips were fixed and permeabilised with 50:50 acetone–methanol and washed with PBS. Transfected cells were identified by expression of EGFP. Primary antibodies were either anti-BioID2 mouse mAb (Abcam plc, Cambridge, UK; ab232733; 2 µg/mL), or anti-Ki67 mouse mAb (Dako, Agilent Technologies Ltd., Stockport, Cheshire, UK; MIB-1) which were detected with secondary antibody goat anti-mouse ALEXA 546 (Invitrogen, Thermo Fisher Scientific, Paisley, UK; Cat No: A11030). Also, the primary antibodies, anti-human PMP22 rabbit mAb (Abcam; Cambridge, UK; ab270400; 1/200~3 µg/mL), anti-ITGA2 rabbit mAb (Abcam; Cambridge, UK; ab181548; 1/400), or anti-ITGA7 rabbit pAb (Invitrogen, Paisley, UK; PA5-49447; 1/25) were detected with secondary antibody goat anti-rabbit ALEXA 546 (Invitrogen; Paisley, UK; Cat No: A11010). Primary antibodies were diluted in PBS and incubated on fixed cells for 1 h. Cells were then washed with PBS and incubated with appropriate secondary antibodies, diluted 1/400 in PBS containing 1% horse serum, 1% foetal bovine serum and 0.1% BSA, for 1 h. Alternatively, to detect biotin, cells cultured in the presence of 50 µM biotin (Sigma-Aldrich, Dorset, UK; B4501) were fixed and permeabilised and incubated with streptavidin ALEXA fluor 555 conjugate (Invitrogen; Paisley, UK; Cat No: S32355; 1/500~4 µg/mL in PBS) for 1 h. To counterstain nuclei, DAPI (200 ng/mL) was added for the final 10 min of incubation, after which the cells were washed and mounted in Dako Fluorescence Mounting Medium (Agilent Technologies UK Ltd., Cheadle, Manchester, UK; Code No: S3023). Processed cells were examined and images acquired by sequential scanning with a Leica TCS SP5 confocal microscope (Objective 63x, NA 1.4; Leica Microsystems, Milton Keynes, UK).

For the quantitation of Ki67, the three cell lines, cultured for 3 days, were processed identically for immunofluorescence staining and the same confocal microscopy settings were used. Image analysis was performed using ImageJ v1.54f, accessed on 21 September 2023 (https://imagej.net/ij/index.html; [30]). DAPI staining was analysed to identify nuclei (>200 µm^2^) and ALEXA 546 staining within nuclei was quantitated as a measure of Ki67 activity. The quantitative measurement of Ki67 was the “mean grey value”, which is the sum of the grey values of all the pixels in a nucleus as a function of area of the nucleus.

For immunofluorescence quantitation of PMP22, the cell lines were cultured for 3 d and 10 d and immunostained identically for PMP22. Identical microscopy and ImageJ settings were used for all cell cultures. Images with just ALEXA 546 were analysed to determine the mean grey value of the whole field of cells. DAPI staining of nuclei was used to determine the total number of cells per field. The mean grey value per cell was calculated by dividing the mean grey value per field by the number of cells per field.

### 2.5. SDS-Polyacrylamide Gel Electrophoresis and Western Blotting

CRL, control-transfected and PMP22-transfected cell lines were pelleted and washed 3 × with Dulbecco’s Phosphate-Buffered Saline (DPBS) (Gibco, Paisley, UK; 14190). Pellets were extracted with RIPA buffer and mixed with 2 × SDS buffer to give final concentrations of 125 mM Tris pH 6.8; 2% SDS; 5% 2-beta mercaptoethanol; 5% glycerol; with bromophenol blue. Samples were boiled and separated by SDS-PAGE on 10% acrylamide gels (Invitrogen; Bolt 10% Bis-Tris Plus; NW00100BOX) and transferred to nitrocellulose membranes (Whatman, Oxfordshire, UK; Protan BA85). Non-specific sites on membranes were blocked with 5% skimmed milk protein and then washed with PBS, prior to incubation for 1 h with primary antibody, which was either anti-BioID2 mouse mAb (Abcam plc, Cambridge, UK; ab232733; 1/1000, 1 µg/mL), anti-GFP mouse mAb (Sigma; G1546; 2 µg/mL), anti-human PMP22 rabbit mAb (Abcam; ab270400; 1/1000~0.6 µg/mL) or anti-ITGA2 rabbit mAb (Abcam; ab181548; 1/1000). Membranes were then washed with PBS and incubated with the appropriate secondary antibody for 1 h, which was either rabbit anti-mouse immunoglobulins HRP (Dako, Glostrup, Denmark; P0260; 1/1000) or goat anti-rabbit immunoglobulins HRP (Dako; P0448; 1/1000). Secondary antibodies were diluted in PBS containing 0.05% Triton X, 0.1% BSA, 1% horse serum and 1% foetal bovine serum, followed by washing with PBS. Peroxidase signals were detected with SuperSignal West Femto chemiluminescent reagent (Thermo Fisher Scientific; 34094) and the ChemiDoc Touch imaging system (BioRad, Watford, UK). For biotin detection, cells were cultured as described in the “immunofluorescence” section and processed as described above, except that the nitrocellulose membrane was probed with streptavidin-HRP (Pierce; 21130; 1/1500,~0.7 µg/mL) diluted in 1% BSA in PBS for 1 h, prior to washing with PBS and detection with SuperSignal West Pico chemiluminescent reagent (Thermo Fisher Scientific; 34580). Densitometry measurements of lanes in Coomassie blue-stained gels, or of bands on nitrocellulose membranes, were determined using ImageJ. Images were converted to grayscale with ImageJ and, within each image, the same size of box was used for measurements of bands or lanes. The area under the intensity curves was measured to give values of relative cumulative intensity.

### 2.6. Myelination Potential Analysis

The three cell lines were passaged at 2 × 10^4^ cells/mL and cultured for 3 days (3 d). The medium was then replaced and culture continued for a further 7 days, either in the absence of additional factors (10 d), or in the presence of 1 nM neuregulin 1 (NRG1; human recombinant heregulin beta 1; Stem Cell Technologies, Cambridge, UK; Cat No: 78071.1; A 65 amino acid peptide containing the 31 amino acid Epidermal Growth Factor-Like domain and flanking regions) and 5 µm forskolin (Stem Cell Technologies; Cat No: 72114) (10 d-treated). Culture conditions to stimulate myelination potential were modified from methods described by others [31,32,33].

### 2.7. RNA Isolation and RT-qPCR

Total RNA was prepared from cultured cells using RNeasy Plus Mini Kit (Qiagen) and quantified by loading samples onto an LVis plate and measuring with a FluoStar Omega plate reader (BMG Labtech Ltd., Ayelsbury, Buckinghamshire, UK). Total RNA (2.5 µg in a 20 µL reaction) was reverse-transcribed using SuperScript VILO cDNA Synthesis Kit (Applied Biosystems, Thermo Fisher Scientific, Paisley, UK). Relative quantitative PCR was performed using SYBR green detection in a QuantStudio 3 real-time PCR system (Applied Biosystems). Reaction wells contained 10 µL SYBR Select Master Mix (Applied Biosystems), 1.5 µL cDNA (equivalent to 10 ng RNA), 300 nM Forward and 300 nM Reverse primers in a final volume of 20 µL. Target sequences were amplified along with two endogenous controls (Beta-actin and GAPDH) and expression of transcripts relative to the two endogenous reference transcripts was calculated by the 2-ΔCT method [34,35]. Primer sequences are shown in Table S1 [20,36,37]. For each data point, results were calculated as the mean (±SD) of at least 3 independent experiments. Groups had approximately equal variance. Data were analysed by two-way ANOVA and Tukey’s multiple comparisons post hoc test, as described earlier.

### 2.8. Cell Extraction and Pull-Down of Biotinylated Proteins

The three cell lines: CRL, control-transfected and PMP22-transfected, were each diluted to 2 × 10^4^ cells/mL in 4 × 20 mL medium in 4 × T75 flasks (Sarstedt, Leicester, UK; 83.3911.002) and cultured for 3 days in the presence of 50 µM biotin (Sigma-Aldrich; B4501). After 3 days in culture, PMP22-transfected cells in flasks were around 70% confluent whereas the other 2 cell lines were 80 to 90% confluent. Flasks were trypsinised and cells from the 4 flasks were combined. The 3 cell pellets were washed 3 × with DPBS on ice, giving pellets of approximately 60 mg (PMP22-transfected) and 70 mg (CRL and control-transfected). Cells were kept on ice and 240 µL RIPA buffer and protease inhibitor cocktail (Sigma, P8340) was added, followed by mixing and sonicating to extract the proteins. Extracts were then centrifuged (12,000 rpm, 20 min) and there were no visible pellets, suggesting that the cells had been extracted efficiently. Dynabeads MyOne Streptavidin C1 superparamagnetic beads (Invitrogen; 65001) in suspension were aliquoted (100 µL per tube) into 3 × Eppendorf tubes. Magnets (Invitrogen; DynaMag Spin, 12320D) were used to concentrate the magnetic beads to the side of the Eppendorf tube to allow aspiration of liquids between washing and incubation steps. The beads were resuspended in liquids by flicking the tubes and were each washed 3 × with DPBS and once with RIPA buffer. Following washing, cell extracts were added to the tubes and incubated with the beads, on a roller, for 2 h, to allow the binding of biotinylated protein. After the incubation step, beads were washed 2 × with RIPA buffer and 4 × with DPBS. Captured proteins were digested by overnight incubation with 1% *v*/*v* sequencing grade trypsin (Promega, Southampton, UK; V5111) at 37 °C. Supernatants containing peptides were then transferred to fresh tubes for analysis.

### 2.9. Mass Spectrometry Analysis of Pull-Downs

Peptides were subjected to LCMS/MS using an Ultimate 3000 RSLC (Thermo Fisher Scientific) coupled to an Orbitrap Fusion Lumos mass spectrometer with a FAIMS interface (Thermo Fisher Scientific). Peptides were injected onto a reverse-phase trap (Pepmap100 C18 5 μm 0.3 × 5 mm) for pre-concentration and desalted with loading buffer (100% water, 0.05% TFA), at 15 µL/min for 3 min. The peptide trap was then switched into line with the analytical column (Easy-spray Pepmap RSLC C18 2 µm, 50 cm × 75 um ID). Peptides were eluted from the column using a linear solvent gradient of buffers A (100% water, 0.1% formic acid) and B (80% acetonitrile, 20% water, 0.1% formic acid) using the following gradient: linear 4–40% of buffer B over 80 min, linear 40–60% of buffer B for 15 min, sharp increase to 95% buffer B within 0.1 min, isocratic 95% of buffer B for 10 min, sharp decrease to 2% buffer B within 0.1 min and isocratic 2% buffer B for 20 min. The FAIMS interface was set to −45 V and −65 V at standard resolution. The mass spectrometer was operated in data dependent acquisition (DDA) positive ion mode with a cycle time of 1.5 s. The Orbitrap was selected as the MS1 detector at a resolution of 120,000 with a scan range from *m*/*z* 375 to 1500. Peptides with charge states 2 to 5 were selected for fragmentation in the ion trap using HCD as collision energy. The raw data files were converted into mgf using MSconvert v3 (ProteoWizard) and searched using Mascot v2.8.1 [38,39], with trypsin as the cleavage enzyme and oxidation as a variable modification of methionines against the Uniprot database, restricted only to proteins from humans (101,676 protein sequences). The mass accuracy for the MS scan was set to 20 ppm and for the fragment ion mass to 0.6 Da.

### 2.10. Ingenuity Pathway Analysis (IPA)

Proteins that were detected following BioID2 pull-down from the PMP22-transfected cells but undetected from the control-transfected and CRL cells were analysed using IPA software v84978992 (Ingenuity Systems, Silicon Valley, CA, USA) [40] to gain insights into the cellular and molecular pathways with which they are associated. A right-tailed Fisher’s Exact Test was used to calculate a *p*-value determining the probability that each cellular and molecular function or canonical pathway assigned to that data set is due to chance alone, and the final list of functions and pathways were ranked accordingly to the resulting *p*-value. Since multiple pathways are tested for each analysis, the Benjamini–Hochberg (B-H) method is used to control the false discovery rate (FDR). The same list of proteins was also subjected to network analysis in IPA. Each identifier was mapped to its corresponding entry in Ingenuity’s Knowledge Base and these proteins were overlaid onto a global molecular network developed from the curated Ingenuity Knowledge Base. Using an algorithm, networks were then generated and assigned scores based on their connectivity. The Functional Analysis of a network identified the biological functions and/or diseases that were most significant to the proteins in the network. A right-tailed Fisher’s Exact Test was used to calculate a *p*-value representing the probability that the overlaps reported between the input data set and the knowledge base pathway/network are due to chance alone. The resulting networks are graphical representations of the molecular relationships between proteins, where proteins are represented as nodes, and the biological relationship between two nodes is represented as an edge (line). All edges are supported by at least one reference from the literature, from a textbook, or from canonical information stored in the Ingenuity Knowledge Base, all supporting an in vivo or in vitro observation of a protein–protein or protein–DNA interaction (as opposed to a merely predicted interaction from in silico experimentation).

## 3. Results

### 3.1. Generation of Stable Cell Lines

A commercially available Schwann cell line was obtained that had been isolated from the sural nerve of a human donor and immortalised by transduction with hTERT (human telomerase reverse transcriptase) and mCdk4 (murine cyclin-dependent kinase) carrying vectors. The sural nerve is a cutaneous sensory nerve, located at the back of the lower leg, which is accessible and commonly biopsied. This has relevance to CMT as there are increased levels of PMP22 in the sural nerve from patients with CMT1A [6] and the cross-sectional area of the sural nerve has been shown to be increased in CMT1A patients compared to controls [31]. Two plasmid expression vectors were used. PMP22 and control (lacking PMP22) expression vectors with the open reading frames EGFP-hPMP22-BioID2 and EGFP-BioID2, respectively, are described in the Methods Section. Plasmid maps show the vector designs, along with an illustration of the recombinant proteins (Figure S1). The immortalised human Schwann cells were transfected with either the PMP22 or control vector and stable transfectants selected by culturing in the presence of geneticin for 8 weeks, and were cloned twice by limiting dilution. Following the second cloning, all the transfected cell populations were observed to express EGFP, indicating they were clonal (Figure S2). Following the initial screen of live cells for EGFP expression, cells were subsequently screened a second time with immunofluorescence staining of the BioID2 tag over a time course of 1, 3 and 5 days since passage. EGFP and BioID2 were colocalised and showed variable expression in the transfectant cell lines (Figure S3).

### 3.2. Cell Line Characteristics

**Figure 1:**
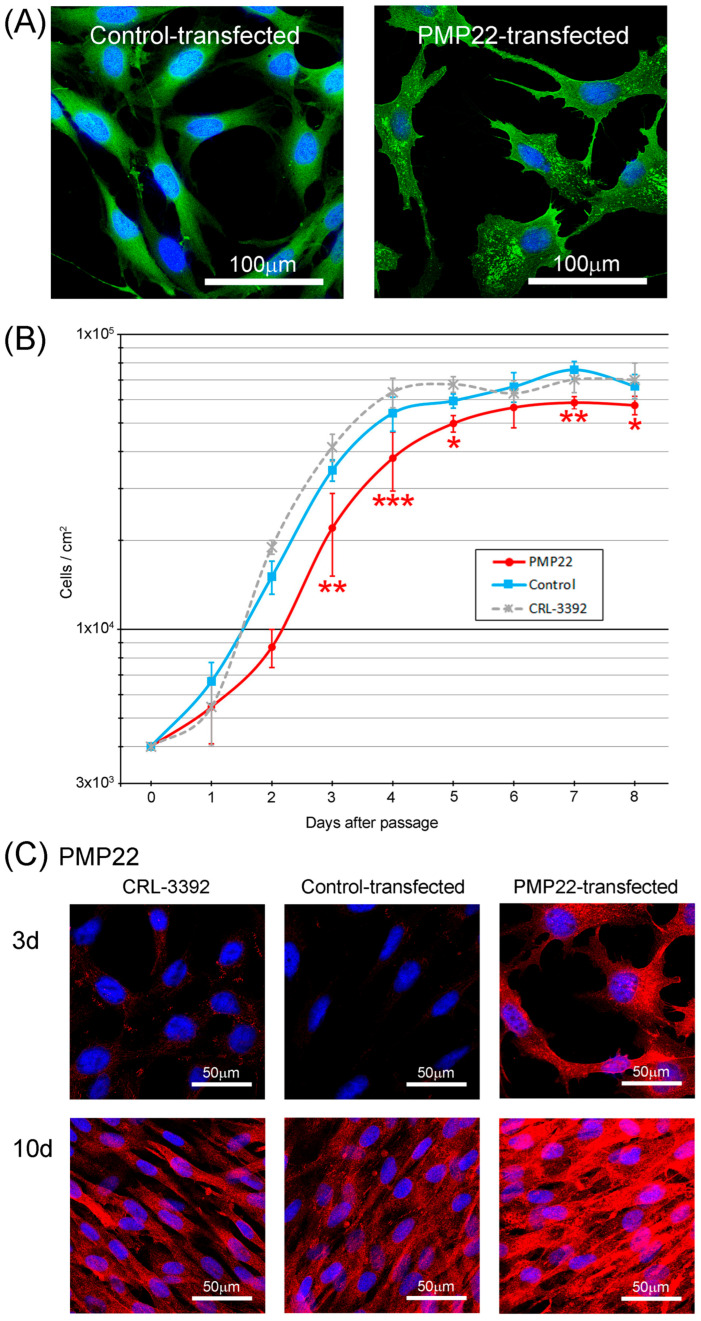
Cell morphology and growth kinetics. (**A**) Control-transfected cells often had smooth plasma membranes and homogeneous expression of EGFP, whereas the plasma membrane of PMP22-transfected cells was more pointed, with punctate aggregates of EGFP concentrating in the cytoplasm. Images consist of EGFP + DAPI (blue). (**B**) Growth curves for the 3 cell lines show exponential growth between approximately 1 and 4 days post-passage, with greater population-doubling time in the PMP22 transfectants. Between 3 and 5 days there were fewer PMP22-transfected cells compared with control and CRL cell lines (*n* = 4 for each cell line at each time point. Two-way ANOVA followed by Tukey’s multiple comparisons post hoc test * *p* < 0.05, ** *p* < 0.01, *** *p* < 0.001). At Day 2 there were fewer PMP22 transfectants compared with CRL cells (*) and at Day 6 there were fewer PMP22 transfectants compared with control-transfected cells (*). (**C**) Immunofluorescence staining for PMP22 in CRL, control-transfected and PMP22-transfected cell lines at 3 days and 10 days post-passage.

The morphology of the PMP22 transfectants was quite striking, with control transfectants often having smooth and regular plasma membrane morphology, which is reminiscent of the parent cell line, whereas PMP22 transfectants had spiky and irregular plasma membranes (Figure 1A). EGFP (transfected material) in control transfectants was homogeneous in the cytoplasm and at the plasma membrane, whereas with PMP22 transfectants, EGFP was again found at the plasma membrane, but also concentrated in punctate aggregates in the cytoplasm, with an often-asymmetric localization (Figure 1A). To determine the growth patterns of the cell lines, T25 flasks were seeded with 2 × 10^4^ cells/mL in 5 mL (4 × 10^3^ cells/cm^2^), and, at 24 h intervals, flasks were trypsinised and cell counts performed. The mean number of cells with an intact plasma membrane (excluding trypan blue) at each time point was determined (Figure 1B). Exponential growth was seen between approximately 1 and 4 days, with cells entering the stationary phase after 4 to 6 days. From 2 to 5 days there were between 1.2 and 2.2 times more non-transfected (CRL) or control-transfected cells compared with the number of PMP22-transfected cells (Figure 1B). During the exponential growth phase, the population-doubling time was 1.3 to 1.4 times greater in PMP22-transfected cells compared with both control-transfected and CRL cells (Figure 1B, Table 1).

To determine whether differences in cell numbers during exponential growth were related to the adherence of the cells, counts of adherent and non-adherent cells were determined at 3 days post passage (Table 1). There were more non-adherent PMP22-transfected cells compared to the other two cell lines (*p* < 0.001), but only 3% of the total PMP22-transfected cells were non-adherent. Non-adherent PMP22-transfected cells accounted for a small part of the differences between adherent PMP22-transfected cell counts and either adherent CRL-3392 (3.6%) or adherent control-transfected cells (5.5%).

To determine whether the difference in cell counts of the three cell lines during exponential growth were due to different frequencies of mitosis, cells were fixed 3 days post-passage and immunostained to detect the nuclear protein Ki67 (Figure S4), which is a graded marker of proliferation. Table 1 shows the quantitation of Ki67 expressed as “mean grey value per nucleus”, which is the sum of the grey values of all the pixels in a nucleus as a function of the area of the nucleus. Nuclear areas in the three cell lines were not significantly different from each other (*p* > 0.3). The quantitation of immunofluorescence in individual nuclei showed 1.5 times more Ki67 in control-transfected compared with PMP22-transfected cells (*p* < 0.001) and 2.5 times more Ki67 in CRL cells compared with the PMP22-transfected cell line (Table 1; *p* < 0.001), which suggests there are significantly reduced levels of mitosis in the PMP22-transfected cells compared to control-transfected and CRL cells during exponential growth.

Levels of PMP22 protein expression of the cell lines at 3 d and 10 d following passage were estimated by immunofluorescence staining (Figure 1C) and quantitation (Table 2).

Results at the two time points show that there is not a constant basal level of PMP22. Endogenous PMP22 in the CRL “parent” cell line at 3 d post-passage is very low. However, when the length of time in culture is increased from 3 d to 10 d, levels of endogenous PMP22 increase more than 4 times in the CRL cell line (*p* < 0.01). As expected, there are higher levels of PMP22 protein in the PMP22-transfected cell line and there is a slight increase in total PMP22 from 3 d to 10 d. PMP22-transfected cells at 3d express around 15 times the level of PMP22 compared to CRL cells at 3 d. However, due to the differences in the levels of endogenous PMP22, PMP22-transfected cells at 3 d express three times the level of CRL cells at 10 d.

**Figure 2:**
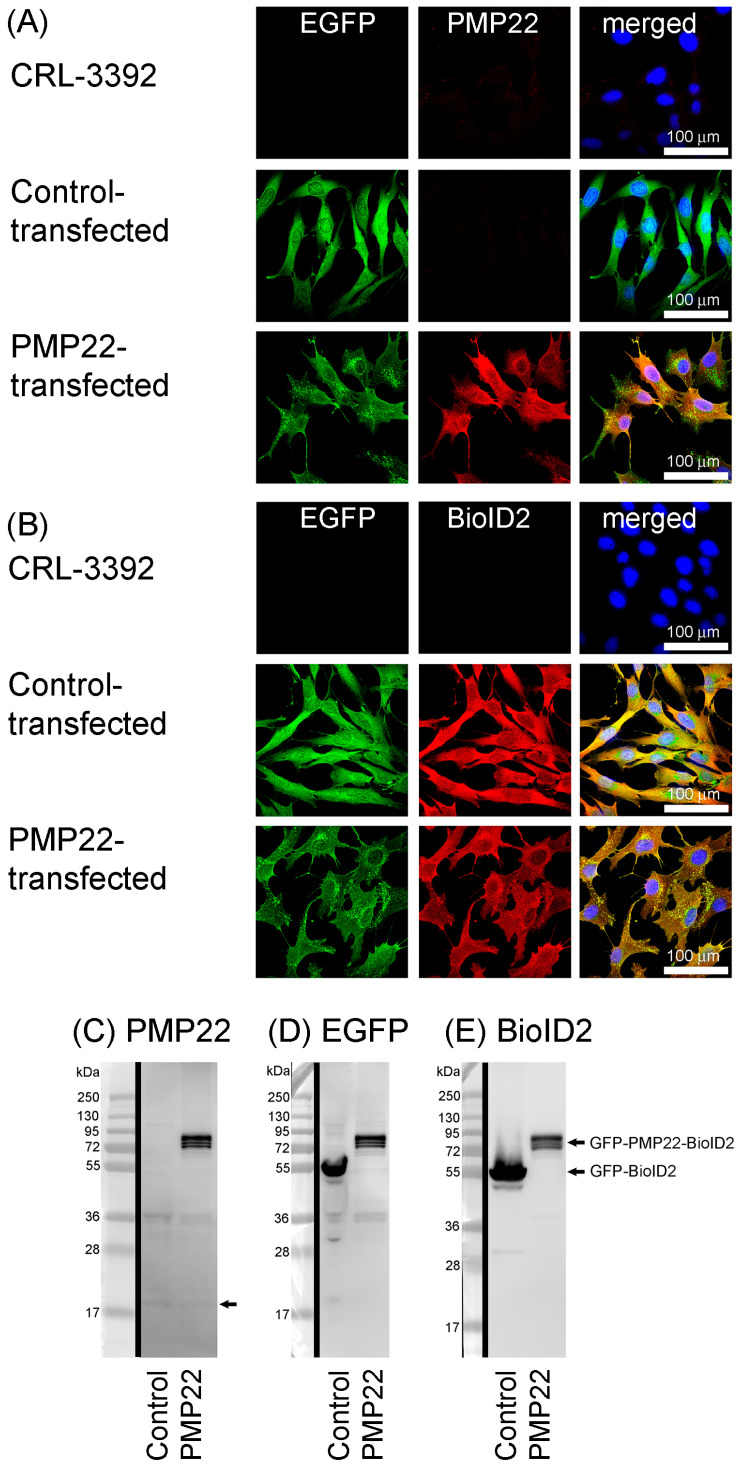
Verification of fusion protein expression. Immunofluorescence analysis showed PMP22 expression in just the PMP22-transfected cells (**A**) and BioID2 expression in both transfected cell lines, but not in CRL cells (**B**). EGFP was observed in both transfected cell lines (**A**,**B**). Merged images show EGFP + PMP22 + DAPI (blue). Similarly, western blot analysis of the two transfected cell lines showed detection of transfected PMP22 in just the PMP22 transfectant (**C**). Weak bands the size of endogenous PMP22 (22 kDa, arrow) were observed in both of the cell lines (**C**). EGFP (**D**) and BioID2 (**E**) were detected in both of the transfected cell lines. The observed molecular weight of bands matched the predicted molecular weights of the EGFP-BioID2 (54 kDa) and EGFP-PMP22-BioID2 (73 kDa) recombinant proteins. Black vertical lines separate the markers (visible light) from the western blot (chemiluminescence).

To verify fusion protein expression, CRL, control-transfected and PMP22-transfected cells were cultured for three days and then fixed and permeabilised for microscopic processing and analysis. As expected, EGFP was present in both transfected cell lines and absent from CRL (Figure 2A,B). As seen previously (Figure 1C), antibody detection of PMP22 protein showed that high levels of PMP22 were present in only the PMP22 transfectants, and were colocalised with EGFP (Figure 2A). Endogenous PMP22 in CRL and control-transfected cells was present at very low levels at this time point. At 3 d, an antibody against BioID2 showed colocalisation with EGFP in both transfected cell lines, but as expected, BioID2 was absent from CRL cells (Figure 2B). Similarly, western blot analysis with an antibody against PMP22 gave a strong band of expected size in just the PMP22 transfectant cell extract (Figure 2C), whereas antibodies against GFP (Figure 2D) and BioID2 (Figure 2E) gave bands of expected sizes in both transfected cell line extracts. Weak bands corresponding to the expected size of endogenous PMP22 monomers were detected in both cell lines (Figure 2C, arrow).

**Figure 3:**
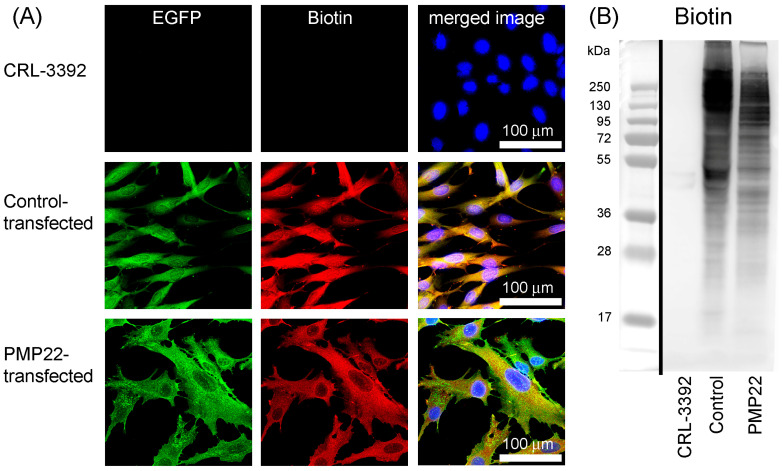
Verification that BioID2 catalyses the biotinylation of endogenous proteins. (**A**) Cells on coverslips were incubated with streptavidin conjugate to confirm that biotinylation had occurred in both transfected cell lines, but not in CRL cells. Merged images show EGFP + biotin + DAPI (blue). (**B**) Electrophoresis of cell extracts and probing with streptavidin-HRP confirmed that biotinylation had not occurred in the CRL cells, but a wide range of molecular weights of biotinylated proteins were present in the control- and PMP22-transfected cell lines. The black vertical line separates the markers (visible light) from the chemiluminescent image.

The BioID2 component of the fusion protein is a ligase tag which catalyses the covalent attachment of biotin to lysine residues. The capacity of the fusion proteins to biotinylate proximal proteins was tested by culturing the cell lines with biotin supplementation (50 µM) for three days. Cells on coverslips were then processed and biotinylated proteins were detected in the two transfectant cell lines colocalised with EGFP, but biotinylated proteins were absent from CRL cells (Figure 3A). Additionally, following the separation of cell extracts by electrophoresis and probing the blotting membrane with streptavidin-HRP, biotinylated proteins spanning a wide range of molecular weights were found in the two transfectant cell lines, but not in CRL cells (Figure 3B).

### 3.3. Myelination Potential of the Cell Lines

Since CMT1A is a demyelinating disease, the myelination potential of the PMP22-transfected Schwann cells and the control-transfected and CRL-parent Schwann cell lines were investigated, which involved culturing the cell lines in the presence of a peptide containing the Epidermal Growth Factor (EGF)-Like domain and flanking regions of neuregulin 1 (NRG1) and also forskolin, using a method modified from that of others [32,33,41]. The transmembrane isoform of NRG1 (type III) is expressed on the surface of axons and the EGF-like domain interacts with ErbB (epidermal growth factor receptor tyrosine kinases) on the membrane of Schwann cells, activating the Phosphatidylinositol 3-kinase (PI3K)/AKT pathway, which promotes myelination [42]. Contact-mediated signalling between axons and Schwann cells is usually required for the development and myelination of the Schwann cells [43]. However, in contrast to transmembrane NRG1 type III, the N-terminal regions of NRG1 types I and II, which contain the EGF-like domain, can be secreted into the extracellular environment as soluble proteins [44]. Soluble NRG1 has been shown to promote myelination in a dose-dependent manner, being inhibitory if the concentration of NRG1 is too high [32].

**Figure 4:**
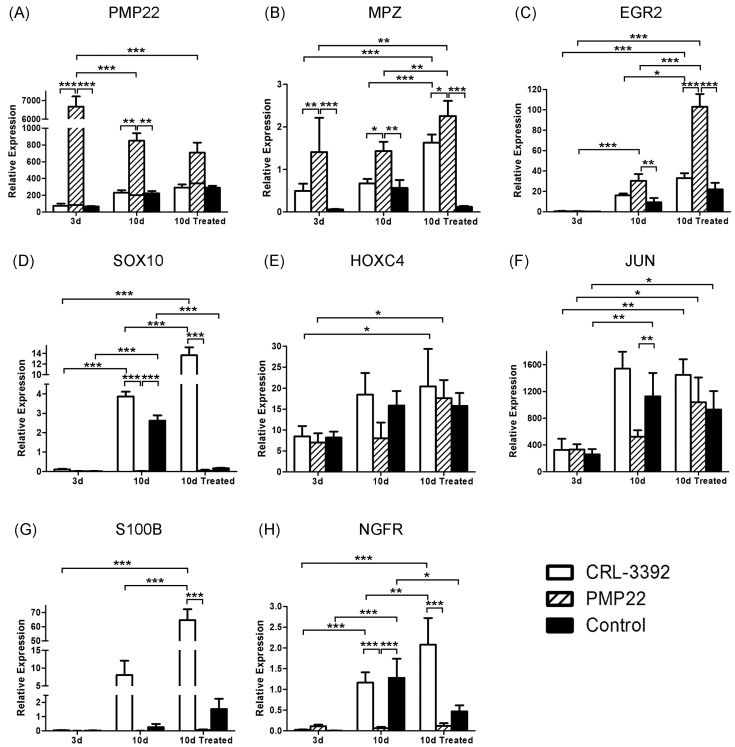
Quantitative PCR to assess myelination potential of the 3 cell lines. Columns represent the mean relative expression and standard deviation of the 3 cell lines (CRL parent cell (clear columns), PMP22-transfected (hatched columns) and control-transfected (solid columns)) at 3 culture time points since passage (3 d, 10 d, 10 d-treated with NRG1 and forskolin). (**A**) Total *PMP22*: In PMP22-transfected cells, total *PMP22* and transfected *PMP22* were measured and results are expressed as a horizontal line within the hatched columns, with the quantity of transfected *PMP22* depicted above the line and endogenous *PMP22* below the line. (**B**) *MPZ*; (**C**) *EGR2*; (**D**) *SOX10*; (**E**) *HOXC4*; (**F**) *c-JUN*; (**G**) *S100B*; (**H**) *NGFR*. Statistical differences between RE values are shown (Two-way ANOVA followed by Tukey’s multiple comparisons post hoc test * *p* < 0.05, ** *p* < 0.01, *** *p* < 0.001). If not shown, there was no significant difference between values (*p* > 0.05).

To attempt to promote pro-myelination potential, NRG1 was initially tested at 0.1 nM, 1 nM and 10 nM in the culture protocol, and it was determined that maximum PMP22 production occurred with 1 nM NRG1 in the CRL-parent cell line in this system. NRG1 was therefore used at 1 nM for all subsequent experiments. NRG1 at 1 nM is comparable to the concentrations used in other systems [32,33,41], whereas 10 nM NRG1 has been shown to inhibit the myelination of rat Schwann cells co-cultured with dorsal root ganglion axons [32]. Forskolin, which induces cAMP and synergistically enhances NRG1 [45], was also included in the pro-myelination cultures. Myelination potential and expression of Schwann cell markers by the cell lines was assessed by passaging and culturing the cells for three days (3 d time point) and then replacing the medium with either fresh medium alone, or with medium containing NRG1 and forskolin (“treated”), to promote myelination potential, and culture was continued for a further seven days (10 d time point). RNA was extracted for qPCR analysis at each time point (3 d, 10 d, 10 d-treated). Measurement of total *PMP22* transcript (Figure 4A) showed that PMP22-transfected cells expressed very high levels of *PMP22* transcript at all time points (relative expression was 6668 ± 555 (3 d), 850 ± 89 (10 d) and 708 ± 119 (10 d-treated)), which at 3 d was 90 to 100 times greater than that of the other two cell lines (*p* < 0.001). At 10 d the level of total *PMP22* transcript in the PMP22-transfected cells was almost four times greater than that of the other cell lines (*p* < 0.001) and at 10 d-treated, total *PMP22* was around 2.4 times greater than the other cell lines (Figure 4A). *PMP22* transcript derived from the transfected vector alone (exogenous) was measured by amplifying with the forward primer within the *PMP22* sequence and reverse primer within the BioID2 sequence, which is the C-terminal component of the fusion protein. The relative expression of exogenous *PMP22* in the PMP22-transfected cells, at the three time points was 6589 ± 377 (3 d), 648 ± 83 (10 d) and 365 ± 101 (10 d-treated). Endogenous *PMP22* transcript in the PMP22-transfected cell line was determined by subtracting exogenous *PMP22* values from total *PMP22* values. In PMP22-transfected cells, the horizontal line in the column chart depicts the value of exogenous *PMP22* above the line and endogenous *PMP22* below the line (Figure 4A). In the three cell lines, there is a 2.6- to 3.3-fold increase in endogenous *PMP22* from 3 d to 10 d and between 10 d and 10 d-treated there is a 1.3- to 1.7-fold increase in endogenous *PMP22*. At individual time points the three cell lines have comparable levels of endogenous *PMP22* (*p* > 0.1) (Figure 4A).

Expression of some myelination markers was decreased and of others increased in PMP22-transfected cells, compared with the other two cell lines (Figure 4). Like PMP22, myelin protein zero (MPZ) is a structural component of myelin. *MPZ* transcript levels increased significantly from 3 d to 10 d-treated in CRL (3.3-fold) and PMP22-transfected cells (2-fold) (Figure 4B). At the three time points, *MPZ* transcript levels were greater in PMP22-transfected cells compared with the other two cell lines (*p* < 0.05) (Figure 4B). Early growth response 2 (EGR2) (Figure 4C) and SRY-Box Transcription Factor 10 (SOX10) (Figure 4D), which are transcription factors that play important roles in Schwann cell differentiation and myelination, and the transcription factor Homeobox C4 (HOXC4; Figure 4E), which may be involved in these processes [19], have different expression profiles. The *EGR2* (Figure 4C) expression profile shares similarities with *MPZ*. Transcript levels of *EGR2* in CRL and PMP22 transfectant cell lines increase from 3 d to 10 d-treated (*p* < 0.001) and from 10 d to 10 d-treated (*p* < 0.05). At 10 d and 10 d-treated, the levels of *EGR2* were 3.1- to 4.7-fold greater in PMP22-transfected cells compared with the other two cell lines (for 10 d-treated, *p* < 0.001; Figure 4C). *SOX10* transcript levels in the CRL cell line increased from 3 d to 10 d and then further increased 3.5-fold at 10 d-treated (*p* < 0.001) (Figure 4D). At 10 d (*p* < 0.001), *SOX10* in PMP22-transfected cells was less than in the other two cell lines, and at 10 d-treated it was less than in the CRL cell line (*p* < 0.001). However, at 10 d-treated, levels of *SOX10* in the two transfected cell lines were both much lower than in the CRL cell line (*p* < 0.001; Figure 4D). *HOXC4* transcript in CRL and PMP22-transfected cell lines increased from 3 d to 10 d-treated (*p* < 0.05). At 10 d, *HOXC4* was 2.0- to 2.3-fold lower in PMP22-transfected cells compared with the other two cell lines (Figure 4E). Jun Proto-Oncogene (JUN) is a component of the AP-1 transcription factor complex and is involved in the Schwann cell response to injury and has variable effects on myelination [46,47,48]. In this system, the expression profile of *JUN* is similar to that of *HOXC4*, showing a general increase from 3 d to 10 d-treated, with *JUN* lower in PMP22-transfected cells (2.2- to 3.0-fold) compared with the control-transfected cell line at 10 d (*p* < 0.01; Figure 4F). S100 calcium binding protein B (S100B) and nerve growth factor receptor (NGFR, also known as neurotrophin receptor P75NTR) both have roles in Schwann cell differentiation and myelination [45,49]. In CRL cells, *S100B* increased from 3 d to 10 d in culture and then increased a further 8-fold from 10 d to 10 d-treated (*p* < 0.001; Figure 4G). Compared with CRL cells, *S100B* in transfected cell lines remained low throughout. *S100B* transcript expression in PMP22-transfected and control-transfected cells were at least 42-fold lower than that seen in CRL cell lines at 10 d-treated (*p* < 0.001; Figure 4G). *NGFR* levels in CRL cells were greater at 10 d and at 10 d-treated compared with at 3 d (*p* < 0.001) and, in control transfectants at 10 d, NGFR was greater compared with at 3 d (*p* < 0.001; Figure 4H). At 10 d and at 10 d-treated, *NGFR* in PMP22-transfected cells was 17-fold lower compared to in CRL cells (*p* < 0.001), but 3.8-fold lower than in control-transfected cells at 10 d-treated (*p* < 0.001; Figure 4H).

### 3.4. BioID2 Identification of PMP22-Associated Proteins in the CMT1A Schwann Cell Model

**Figure 5:**
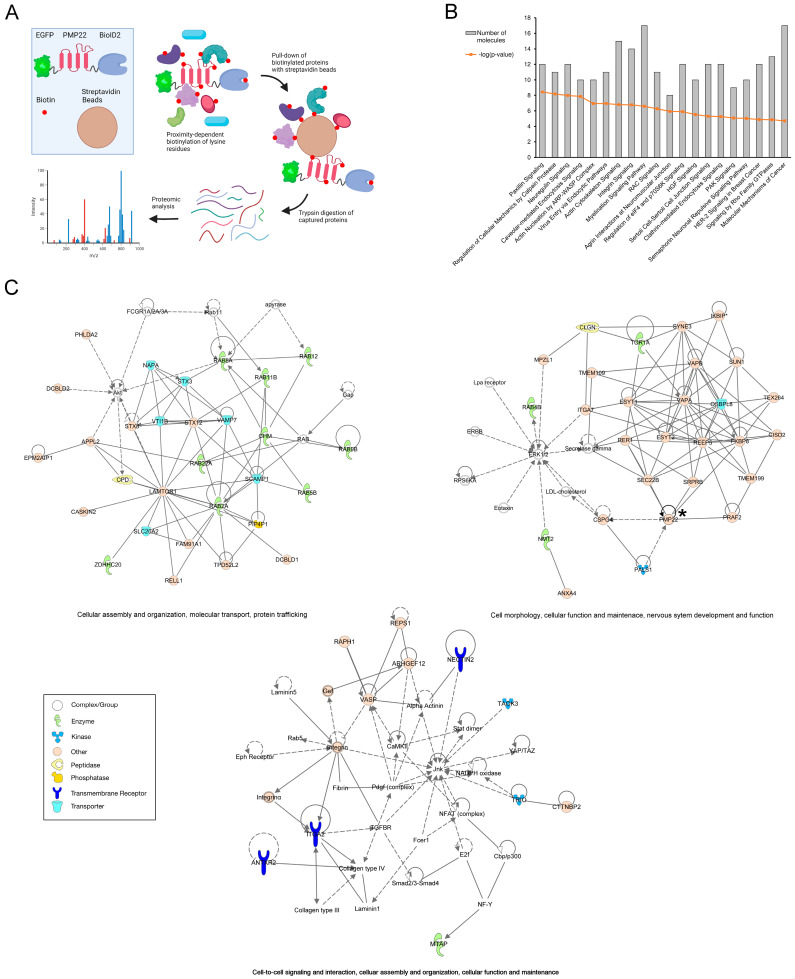
BioID2 method for the identification of PMP22-associated proteins in Schwann cell cultures. (**A**) A schematic representation of the proximity-dependent biotinylation and pull-down method. Recombinant PMP22 contains an N-terminal BioID2 tag which catalyses the biotinylation of proteins that are interacting with, or in the proximity of the recombinant protein. Streptavidin beads were used to pull-down biotinylated proteins, which were subsequently digested with trypsin and analysed by mass spectrometry. Image created with Bio Render.com (https://www.biorender.com). (**B**) Enriched canonical pathways in PMP22-transfected cells, identified by Ingenuity Pathway Analysis (IPA). (**C**) Networks generated by IPA of proteins only detected in the pull-downs from the PMP22-transfected sample.

Proximity-dependent biotin identification (BioID) is a powerful tool for identifying protein–protein interactions and associations in live cells by catalysing covalent bond formation between biotin and lysine residues on interacting and proximate proteins [50]. Here, we employed the BioID methodology to identify proteins in proximity to PMP22 in our newly established Schwann cell model of CMT1A, with the aim of revealing insights into the molecular processes involved in CMT1A pathogenesis and of identifying potential targets for a therapy design aimed at reducing the aberrant accumulation of PMP22. A schematic representation of the proximity-dependent method is summarised in Figure 5A and described in “Materials and Methods”. The smaller ligase that we have used, BioID2, gives enhanced efficiency of labelling, and flexible linkers reduce the steric hindrance to increase the biotinylation range (>10 nm) [51]. For these experiments, the cell lines were passaged and cultured for three days prior to processing for mass spectrometry.

BioID2 pull-down followed by mass spectrometry analysis identified 291 proteins from two or more significant peptides in the PMP22-transfected Schwann cells that were undetected in pull-downs from the control-transfected and CRL cells. Unfiltered mass spectrometry results are shown in Supplementary File S1 and the proteins identified from the greatest number of peptides in just the PMP22-transfectants are shown in Table 3.

PMP22 itself was detected only in the PMP22-transfected cells, but from just one significant peptide, presumably due to the masking of trypsin cleavage sites and/or the addition of unexpected masses arising from the BioID2 and EGFP tags. Pals1, a known trafficker of PMP22 [52] was identified in only the PMP22 pull-down from two significant peptides, providing robust support that this is a valid approach to finding PMP22-associated proteins. It should be noted, however, that by excluding proteins that were also detected in the control-transfected and CRL pull-downs, some genuine PMP22-associated proteins may have been inadvertently overlooked due to their propensity to also bind non-specifically to the streptavidin beads. An enrichment analysis, for example, revealed a greater number of peptides detected from stromal interaction molecule 1 (STIM1), the Ca^2+^ sensor SOC channel subunit in the ER known to co-immunoprecipitate with PMP22 in rat sciatic nerve extracts [53], in the PMP22-transfected sample (*n* = 10 peptides) compared to the CRL sample (*n* = 5 peptides) and control-transfected sample (*n* = 0 peptides).

The PMP22-interacting protein with the highest number of significant sequences and which was not detected in the other cell lines was extended synaptotagmin-1 (ESYT1) variant 2 (Table 3). Commercial antibodies are unable to distinguish ESYT1 variants 1 and 2, although immunolocalization with a pan-ESYT1 antibody revealed some close associations with transfected PMP22, but with little direct colocalisation (Figure S5). ESYT1 is a component of the endoplasmic reticulum membrane, and much of the transfected PMP22 appeared to localise within the endoplasmic reticulum and also at the plasma membrane. Only one peptide in the analysis was unique to ESYT1 variant 2, however, with other peptides also found in the canonical form, ESYT1 variant 1, which was detected in all three of the pull-downs. As there was only one unique variant 2 peptide in the PMP22 pull-down, it is possible that ESYT1 variant 2 is also present in the other two pull-downs, but undetected.

Ingenuity Pathway Analysis (IPA^®^) of the proteins that were only detected in the pull-down from the PMP22-transfected sample and undetected in the control-transfected and untransfected samples revealed several enriched canonical pathways: paxillin signalling (*p* = 3.57 × 10^−9^), regulation of cellular mechanics by calpain protease (*p* = 6.45 × 10^−9^), neuregulin signalling (*p* = 9.97 × 10^−9^), and caveolar-mediated endocytosis signalling (*p* = 1.35 × 10^−8^) (Figure 5B). Subsequent IPA^®^ network analysis of the proteins pulled down in only the PMP22-transfected sample identified 16 networks with which the proteins were associated. The top two ranking networks were associated with “cellular assembly and organisation, molecular transport and protein trafficking” and “cell morphology, cellular function and maintenance, nervous system development and function”, with PMP22 itself being associated with the latter (Figure 5C). A separate, but similarly themed network, “cell-to-cell signalling and interaction, cellular assembly and organisation, cellular function and maintenance” was identified which included the second-most enriched protein from the PMP22 pull-down, the transmembrane receptor, integrin alpha 2 (ITGA2), along with two other transmembrane receptors (Figure 5C).

**Figure 6:**
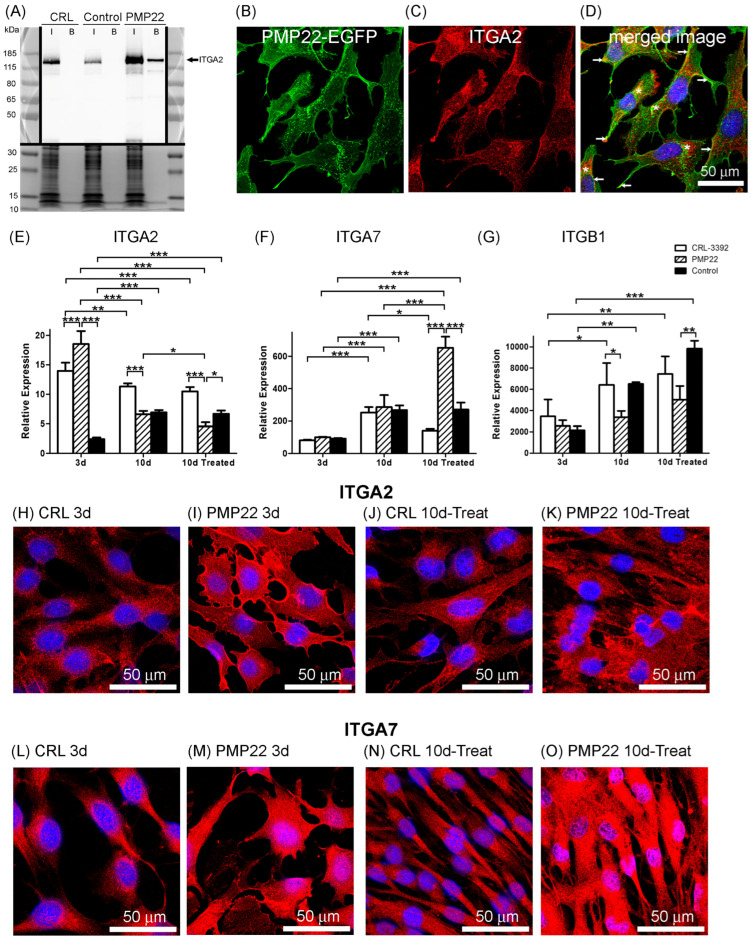
Integrin expression in Schwann cell lines. (**A**) When cultured for 3 days, western blot analysis of BioID2 pull-downs of the three cell lines confirmed that ITGA2 (predicted molecular weight 129 kDa) was present in the “Input” samples but was “Bound” only in the PMP22 transfectants. The lower part of the gel was stained with Coomassie blue to show approximately equal levels of protein in the three “Input” samples, with no low molecular weight proteins observed in the “Bound” fractions. Black vertical lines separate the markers (visible light) from the western blot (chemiluminescence). Below the black horizontal line, the stained gel was detected with visible light. When cultured for 3 days, immunofluorescence analysis showed some colocalisation between transfected PMP22 (EGFP) (**B**) and ITGA2 (**C**) in the merged image (EGFP + ITGA2 + DAPI (blue)) (**D**). Colocalisation was seen in the cytoplasm (white stars) and at the plasma membrane (white arrows) in the merged image of PMP22-transfected cells (**D**). Quantitative PCR of integrin transcript expression in the 3 cell lines. Columns represent the mean relative expression and standard deviation of the 3 cell lines (CRL-parent cell (clear columns), PMP22-transfected (hatched columns) and control-transfected (solid columns)) at 3 culture time points since passage (3 d, 10 d, 10 d-treated with NRG1 and forskolin). (**E**) *ITGA2*; (**F**) *ITGA7*; (**G**) *ITGB1*. In PMP22-transfected cells, relative expression of *ITGA2* was greatest at 3 d and of *ITGA7* greatest at 10 d-treated. Statistical differences between RE values are shown (Two-way ANOVA followed by Tukey’s multiple comparisons post hoc test * *p* < 0.05, ** *p* < 0.01, *** *p* < 0.001). If not shown, there was no significant difference between values (*p* > 0.05). Representative immunofluorescence analysis of ITGA2 (**H**–**K**) and ITGA7 (**L**–**O**) in CRL and PMP22-transfected cell lines, at 3 d and 10 d-treated. Localisation in CRL cells was very similar to that seen with control-transfected cells. In PMP22-transfected cells, the highest levels of ITGA2 were seen at 3 d at the plasma membrane (**I**), whereas the highest levels of ITGA7 were seen throughout the cells at 10 d-treated (**O**).

Western blot analysis (at 3 d) confirmed that ITGA2 was detected in all three of the input samples but was bound only in the PMP22 pull-down (Figure 6A; Densitometry measurements shown in Supplementary File S3). Coomassie staining of the lower part of the gel showed similar levels of protein in the three input samples (Figure 6A). Immunolocalization at 3 d revealed some colocalisation between transfected PMP22 and ITGA2 in the cytoplasm and concentrated at the plasma membrane of PMP22-transfected cells (Figure 6B–D). Another integrin, integrin alpha-7 (ITGA7) was also detected in just the PMP22 pull-down (Table 3).

### 3.5. Integrins and Myelination

At 3 d, ITGA2 and ITGA7 were detected by mass spectrometry with high significance in just the PMP22 pull-down (Table 3). Integrins are transmembrane cell-adhesion proteins that usually exist as heterodimers at the plasma membrane. ITGA2 and ITGA7 both partner integrin β1 (ITGB1) and bind components of the extracellular matrix, and the interaction of Schwann cells with the extracellular matrix is required for correct myelination to occur. To further study the relationships between integrins and myelination, cDNA preparations from the Schwann cell cultures described earlier (3 d, 10 d and 10 d-treated; Figure 4) were analysed by qPCR to determine the relative expression of *ITGA2*, *ITGA7* and *ITGB1* transcripts (Figure 6E–G). At 3 d, the relative expression of the *ITGA2* transcript was greater in the PMP22 transfectants compared to the other two cell lines (*p* < 0.01) (Figure 6E), which was also seen with total *PMP22* (Figure 4A). At 10 d and 10 d-treated, *ITGA2* levels were lower in both PMP22-transfected (*p* < 0.001) and CRL (*p* < 0.005) cell lines compared to those at 3 d (Figure 6E). At 10 d *ITGA7* levels were increased in all three cell lines, compared to the levels at 3 d (*p* < 0.001). The *ITGA7* transcript in PMP22-transfected cells at 10 d-treated was two- to five-times greater than the other cell lines at 10 d-treated (*p* < 0.001) and seven-times greater than all cell lines at 3 d (*p* < 0.001; Figure 6F). ITGB1 was not detected by mass spectrometry in the pull-down at 3 d, but levels of *ITGB1* transcripts were determined, because ITGB1 forms dimers with both ITGA2 and ITGA7. *ITGB1* transcript was detected at high levels and there was a general increase in *ITGB1* from 3 d to 10 d-treated in the CRL and control-transfected cell lines (*p* < 0.01). Levels of *ITGB1* transcript in PMP22-treated cells were lower than in CRL cells at 10 d (*p* < 0.05) and lower than in control-transfected cells at 10 d-treated (*p* < 0.01) (Figure 6G). The relative expression levels of the three integrin transcripts indicates that *ITGB1* was in great excess compared to the other two integrins (Figure 6E–G), possibly reflecting the multiple functions of ITGB1. For example, for CRL cells at 10 d, the relative expression levels of *ITGA7* are 22-times greater than *ITGA2*, and the relative expression levels of *ITGB1* are 25-times greater than *ITGA7*.

CRL and PMP22-transfected cell lines on coverslips cultured for 3 d, 10 d and 10 d-treated, were immunostained for ITGA2 (Figure 6H–K) or ITGA7 (Figure 6L–O). At 3 d, ITGA2 in CRL cells was localised mainly in the cytoplasm (Figure 6H), whereas, as seen previously at 3 d (Figure 6C), ITGA2 in PMP22-transfected cells was found concentrated in regions of the plasma membrane, with lower levels in the cytoplasm (Figure 6I). When myelination potential was stimulated (10 d-treated), the majority of ITGA2 in CRL cells appeared to remain in the ER, with some also localising to the plasma membrane (Figure 6J), and, in PMP22-transfected cells, ITGA2 localised to the cytoplasm, with some remaining concentrated in regions of the plasma membrane (Figure 6K). At 3 d, ITGA7 in CRL cells was mainly cytoplasmic (Figure 6L) and ITGA7 in PMP22-transfected cells was localised throughout the cells (Figure 6M). The stimulation of myelination potential led to the expression of ITGA7 in the cytoplasm and at the plasma membrane of CRL cells (Figure 6N) and high levels of ITGA7 throughout PMP22-transfected cells (Figure 6O). Immunofluorescence and qPCR results in this culture system indicated that, in PMP22-transfected cells, the highest levels of ITGA2 transcript and protein occurred at 3 d, whereas the highest levels of ITGA7 transcript and protein occurred whilst myelination potential was being stimulated at 10 d-treated (Figure 6).

**Figure 7:**
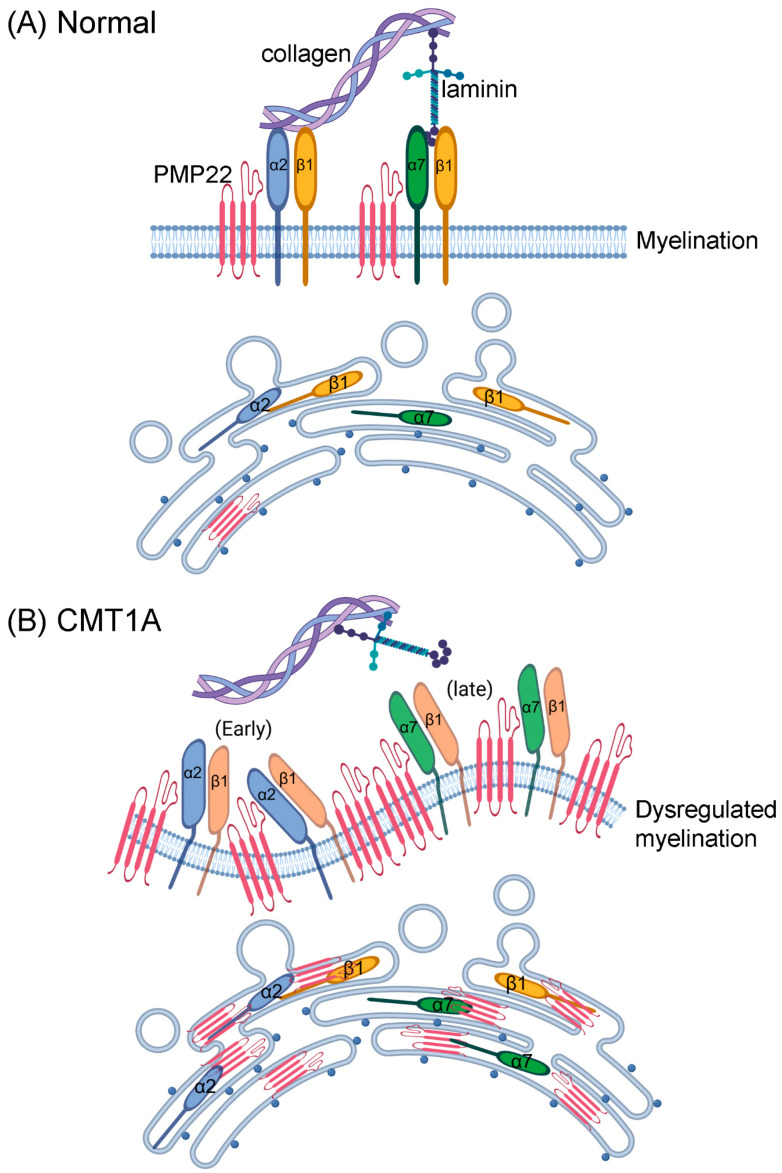
Schematic diagram to illustrate possible relationships between PMP22 and integrins in (**A**) normal and (**B**) CMT1A Schwann cells. (**A**) In normal Schwann cells, integrins and PMP22 are processed in the endoplasmic reticulum and Golgi apparatus, from where they are transported to the plasma membrane. Integrins form dimers, which, when in the correct conformation, can interact with components of the extracellular matrix. Integrins α2β1 connect to collagen and laminin and integrins α7β1 connect to laminin. PMP22 is required for the accurate interaction of Schwann cells with the extracellular matrix, which is required for correct myelination in the peripheral nervous system. (**B**) In CMT1A Schwann cells, excessive PMP22 is produced, which may lead to oversaturation of the ER quality control network and the accumulation of misfolded PMP22 protein in the ER. Increased production of ITGA2 and ITGA7 also occurs. Whilst the ER becomes saturated, high levels of PMP22 and integrins may also be expressed at the plasma membrane. The mechanism(s) by which PMP22 saturation disrupts interaction with the extracellular matrix has yet to be elucidated. qPCR and immunofluorescence microscopy indicate that in PMP22-transfected cells maximum expression of ITGA2 is an early event whereas maximum expression of ITGA7 occurs later in the myelination process (see Figure 6). Much of the excess PMP22 may be largely misfolded and not function correctly. Saturation of the ER with PMP22 may interfere with the normal processing and trafficking of integrins. An excess of PMP22 at the plasma membrane may inhibit the binding of integrins to the extracellular matrix. Disruption of the interaction between Schwann cells and the extracellular matrix leads to defective/dysregulated myelination. Image created with Bio Render.com (https://www.biorender.com).

Figure 7 illustrates a possible mechanism whereby integrins on the plasma membrane of Schwann cells interact with the extracellular matrix in normal myelinating Schwann cells, whereas in CMT1A overproduction of PMP22 is associated with increased production of ITGA2 (early response) and ITGA7 (later response). It could be speculated that high levels of PMP22 may interfere with the interaction between integrins and the extracellular matrix, and so lead to defective or dysregulated myelination.

## 4. Discussion

### 4.1. Overexpression of PMP22

Here, we have described the development of a clonal, immortalised, stably transfected human Schwann cell model of CMT1A that overexpresses PMP22 recombinant fusion protein, which contains an EGFP tag for the visual identification of transfectants and a biotin ligase (BioID2) tag for proximity-dependent biotin identification. We have also produced a control cell line expressing a recombinant protein containing the two tags alone. We found that much of the overexpressed PMP22 fusion protein localised as intracellular aggregates, with some present at the plasma membrane. Similar intracellular aggregates of PMP22 have been observed by others, for example, in human CMT1A Schwann cells [54] and human fibroblasts [55], in rat models [56], in mouse models [57,58] and in transient transfection models [29]. Protein aggregates are also seen in the cytoplasm of Schwann cells from Trembler J mice, which contain a missense mutation in PMP22 [59,60,61]. At 10 d the level of total *PMP22* transcript in the PMP22-transfected cells was almost four-times greater than that of the other cell lines and at 10 d-treated, *PMP22* transcript was more than two-times greater in PMP22-transfected cells compared with the other cell lines. This aligns with previous findings where normalised *PMP22* transcript levels in dermal nerves were very variable but elevated up to six-fold in CMT1A patients compared with controls [62]. Other studies have also shown highly variable levels of PMP22 transcript and protein in CMT1A, which are sometimes in the normal range [42], and it should be noted that it is difficult to gain an understanding of in vivo levels from the limited literature that is currently available.

The relevance to CMT1A of the level of PMP22 expressed by the transfected cells is not straightforward to determine. CMT1A is caused by the duplication of the *PMP22* gene, resulting in three copies instead of two, and so an approximately 1.5-fold increase in PMP22 protein in CMT1A may be expected; however, very little quantitative data is available. In these cell lines, following passage, the baseline production of endogenous PMP22 is variable at different lengths of time in culture. Here, immunofluorescence measurements indicate that PMP22-transfected cells at 3 d express around 15-times the level of PMP22 compared to CRL cells at 3 d, which may be supraphysiological and may be a limitation of the model that should be taken into account. However, PMP22-transfected cells at 3 d express around 3-times the level of CRL cells at 10 d, which may be in the range seen in CMT1A. These differences illustrate the difficulties when trying to assess the physiological relevance of cell models. Mass spectrometry analysis was performed at 3 d post-passage, with the cells undergoing exponential growth. This time point was chosen because the cells were in a state of active proliferation, with minimal influences from nutrient depletion, contact inhibition and senescence, which are likely to occur more as proliferation continues.

### 4.2. Myelination Potential of Schwann Cell Lines

In the present study, at three days post-passage, low levels of endogenous *PMP22* transcript were observed in the cell lines. However, increasing the length of post-passage days in culture up to 10 d led to increased levels of *PMP22*. Other markers of myelination often also showed increased levels from 3 d to 10 d (Figure 4). The stimulation of myelination potential in the CRL-parent Schwann cell line with NRG1 and forskolin (10 d-treated) led to elevated levels of *MPZ*, *EGR2*, *SOX10*, *HOXC4*, *JUN*, *S100B* and *NGFR* compared with untreated CRL cells (3 d), and elevated levels of *MPZ*, *EGR2*, *SOX10*, *S100B* and *NGFR* compared with untreated CRL cells at 10 d, indicating that the treatment method successfully stimulated myelination potential in these cells. This general increase, however, was not seen for all myelination markers in all three cell lines (Figure 4). The control-transfected cell line showed decreases in *SOX10* and *NGFR* from 10 d to 10 d-treated. Also, at 10 d-treated, *MPZ*, *SOX10*, *S100B* and *NGFR* were much lower in control-transfected cells compared with CRL cells. The findings suggest that stable transfection with the control plasmid led to a reduced expression of some transcripts of myelination potential markers under these conditions.

Some markers of myelination (*SOX10*, *HOXC4*, *JUN*, *S100B* and *NGFR*) were lower in PMP22-transfected cells compared with the other cell lines (at 10 d and/or 10 d-treated), which is consistent with the defective myelination seen in CMT1A. However, there were also occasions when marker levels (*MPZ* and *EGR2*) were raised to a greater extent in PMP22-transfected cells compared with the other cell lines following the induction of myelination potential. The increased levels of *MPZ* and *EGR2* in the PMP22-transfected cells may indicate an adaptive response or compensatory mechanism of the Schwann cells as they attempt to correct the myelination defects, in view of what is known about the complex balance between positive signals and negative regulators during myelination in CMT1, in terms of inefficient myelination or remyelination [63]. The transmembrane protein MPZ is the most abundant myelin protein in the peripheral nervous system, and is involved in maintaining the stability of compact, stacked myelin membranes [64], and mutations in *MPZ* cause CMT1B [19]. *EGR2* encodes a zinc finger transcription factor that upregulates myelination markers, and overexpression of EGR2 increases myelination frequency [65]. Mutations in *EGR2* also cause a type of CMT, CMT1D [19].

The finding that other transcripts of markers of myelination were reduced in expression in the PMP22-transfected cells suggests that myelination potential is perturbed in the presence of high levels of PMP22 in both normal culture conditions and when myelination potential is promoted by the addition of forskolin and NRG1. SOX10, for example, which was reduced in PMP22-transfected cells in both conditions compared to CRL cells, is a transcription factor that is crucial for the differentiation and maintenance of Schwann cells and promotes myelination [49]. Similarly, S100B, a calcium-binding protein which is expressed in several cell types, including Schwann cells, was reduced in 10 d-treated PMP22-transfected cells compared to CRL cells. This may be expected, however, since S100B expression is induced by SOX10, which increases Schwann cell proliferation, migration and myelination [49]. Effects of S100B are dependent upon its levels; low levels of S100B may promote myelination whereas high levels may be toxic. NGFR is a cell surface receptor for nerve growth factor (NGF) and is a Schwann cell marker of myelinating phenotype [45]. Reduced levels of *NGFR* in PMP22-transfected cells compared to controls further supports the idea that PMP22 overexpression disrupts the myelination potential of Schwann cells. Patient-specific hiPSCs have been used as a model system [20] and, unlike control hiPSCs, CMT1A hiPSCs were severely defective in their ability to differentiate into Schwann cells through neural crest stem cells (NCSCs). Using immunostaining, S100B and other markers were strongly expressed in control, but not in CMT1A cultures, and transcripts including *SOX10*, *S100B* and *HOXC4*, a transcription factor that is expressed in the neural crest [66] and in Schwann cells [20], were higher in control compared with CMT1A Schwann cells [20], which is similar to the findings of the present study (Figure 4). Following treatment to induce myelination potential, expression of *HOXC4* and *JUN* in PMP22-transfected cells was similar to that seen in the other two cell lines. JUN is a transcription factor subunit and has a complex role in myelination [46,47,48]. Expression of JUN has been shown to inhibit myelination, and while JUN is downregulated in myelinating Schwann cells, its expression is increased following nerve injury and augments the remyelination response [48,67]. JUN was raised in uninjured Schwann cells from a mouse model of CMT1A, but levels were not so high as to disrupt myelination [47]. It has been suggested that boosting JUN expression in CMT1A may reduce axonal loss, but this would need to be carefully controlled due to the potentially demyelinating effect of high levels of JUN [48].

### 4.3. Proximity Analysis of Overexpressed PMP22

The sensitive pull-down in this study is based on the proximity-dependent biotin identification (BioID) method [68], where the targeting protein contains a promiscuous biotin protein ligase tag which covalently attaches biotin to lysine residues in living cells. The practical labelling radius of BioID has been estimated to be around 10 nm [50]. In the present study we have used the smaller and more efficient biotin ligase, BioID2, and have used flexible linkers to separate the different components of the fusion proteins, to overcome any potential spatial constraints [51]. These adaptions are likely to alleviate any steric hindrance and increase the labelling radius of the biotin ligase [51]. The 3x(GGGGS) flexible linker used to separate BioID2 and PMP22 is around 5.7 nm in length [69]. Following the BioID2 pull-down method, IPA bioinformatic analysis revealed enriched functional networks in the pull-downs from PMP22-transfected cells relevant to CMT1A, including: “cell morphology, cellular function and maintenance, nervous system development and function”, which includes PMP22 in the network, along with ESYT1 (component of the ER membrane), ITGA7 (transmembrane receptor), PALS1 (cell polarity protein) and LDL-cholesterol. In this respect, PMP22 is involved in metabolism of the lipid cholesterol, which is an essential component of myelin, and aberrant cholesterol metabolism has been implicated in demyelination [70]. The pull-down protein with the highest number of significant sequences which was detected only in the PMP22-transfected cell line was ESYT1 variant 2. ESYT proteins localise to the ER membrane and are involved in tethering the ER to the plasma membrane and to mitochondria and in the transfer of lipids between adjacent membranes [71]. Endoplasmic reticulum-associated degradation (ERAD) of PMP22 is a normal function of the ER in Schwann cells [4]. The overexpression of PMP22 in CMT1A may lead to the accumulation of cytotoxic aggregates in the ER and Schwann cell dysfunction [10]. Any role of ESYT proteins in these processes has yet to be elucidated. Immunolocalisation showed a close association but little direct colocalisation between pan-ESYT1 and transfected-PMP22 (Figure S5). An antibody specific for ESYT1 variant 2 was not available.

A recent study has described the transcriptomic changes in patient-derived induced pluripotent stem cells differentiated into Schwann cell precursors (iPSC-SCPs) [22]. The iPSC cultures were generated from fibroblasts from a CMT1A patient, which were differentiated into Schwann cell precursors. During their differentiation protocol, they found increased levels of transcripts of markers of Schwann cell development, including *MPZ*, *SOX10* and *NGFR*, between days 0 and 28 [22]. In the present study, we stimulated myelination potential from 3 days to 10 days and also found increased transcripts of *MPZ* (in all three cell lines) and *SOX10* and *NGFR* (in CRL and control cell lines) (Figure 4). They found that Schwann cell development, myelin-related, lipid metabolism and autophagy genes were dysregulated in the CMT1A cell line compared to the control [22]. Of the 24 transcripts described, only PMP22 was represented in the 291 significant proteins detected only in the PMP22 pull-down in the present study (Supplementary File S1). Five proteins in the CRL pull-down (Supplementary File S1) had dysregulated transcripts in the iPSC-SCPs study [22]. It is widely observed in published studies that proteome and transcriptome studies rarely align, even from the same samples, and that this is likely a biological phenomenon, rather than a technological limitation [72]. This likely explains some of the differences observed here at the protein level compared to transcriptome changes in the previously published iPSC-SCP study [22]. In addition, for the transcriptome study, bulk RNA sequencing of the iPSC-SCPs was performed, whereas in the present study, following pull-down, proteins were identified by mass spectrometry, so that only proteins interacting with or in close proximity to the overexpressed PMP22 protein were detected. Similarities between the two studies include the Ingenuity Pathway Analysis in the present study, which identified “lipid metabolism” in the enriched biofunctions category and “myelination signalling pathway” and “autophagy” in the enriched canonical pathways category (Supplementary File S1), with these related genes being dysregulated in the CMT1A iPSC-SCPs [22].

### 4.4. Mitosis in Schwann Cell Lines

Alterations to cellular function and maintenance, which typically includes cell cycle regulation as a major aspect, were also evident when we measured Ki67 levels in PMP22-transfected cells. During exponential growth, PMP22-transfected cells contained lower levels of Ki67 compared to both control and CRL cells, indicating that overexpression of PMP22 is associated with reduced mitosis. During interphase, Ki67 localises to nucleoli and associated structures and plays a role in nuclear organisation and replication timing [73]. Ki67 is continuously produced during the cell cycle from the start of S phase until the exit from mitosis, and is continuously degraded during G0 and G1 [74]. Levels of Ki67 are therefore highly dependent on the current cell cycle phase and are also a function of previous cell cycle activity. When re-entering the cell cycle, a cell that has spent a long time in quiescence will contain lower levels of Ki67 compared to a cell that has spent a shorter time in quiescence [74]. Finding reduced levels of Ki67 in the PMP22-transfected cells is in agreement with an earlier study which showed that mitosis was reduced in dermal fibroblasts from CMT1A patients [55].

### 4.5. Integrins in CMT1A

Another enriched network that was identified among the proteins pulled down from PMP22-transfected cells was “cell to cell signalling and interaction, cellular assembly and organisation, cellular function and maintenance”, which includes transmembrane receptors, including ITGA2, that mediate the interaction between Schwann cells and the extracellular matrix. In this Schwann cell model, protein proximity labelling demonstrated that overexpressed PMP22 interacted with integrins α2 (ITGA2) and α7 (ITGA7), with the ITGA2 interaction verified by western blot analysis. Integrins are type I transmembrane cell-adhesion proteins that usually exist as heterodimers, composed of an alpha subunit and a beta subunit, at the plasma membrane. The alpha subunit of both ITGA2 and ITGA7 partner β1, with α2β1 binding collagen and α7β1 binding laminin [75]. This association was also demonstrated in the network analysis of the proteins pulled down from the PMP22-transfected sample, which highlighted an enriched network containing ITAG2, collagen and laminin. Integrins contain large extracellular domains and short cytoplasmic domains that bridge the extracellular matrix to the intracellular cytoskeleton and are required for several processes including cell adhesion, migration and signal transduction [76,77]. The interaction of integrins with their ligands is highly dynamic and may be regulated by conformational changes which create or expose binding sites for effector molecules [77]. Integrins play a vital role in the interaction of Schwann cells with the extracellular matrix, which, along with the interaction with axons, is essential for correct myelination in the peripheral nervous system [78]. It has been shown, for example, that Schwann cells of ITGB1-null mice do not form normal processes around axons, and these animals develop severe neuropathy [79]. In a mouse model in which laminin-ITGB1 signalling was inhibited and defective myelination occurred, myelination was improved by increasing laminin-ITGB1 signalling at the Schwann cell membrane [80].

PMP22 is required for normal nerve development, as shown by the myelin abnormalities in PMP22-deficient mice [81]. Interaction between ITGB1 and epithelial membrane protein-2 (EMP2), which is a PMP22 family member, has been demonstrated [82]. PMP22 protein has been shown to interact with α6β4 integrin and play a role in mediating the interaction of Schwann cells with the extracellular matrix [83]. In addition, integrin α7β1 has been shown to be required for normal axon development [84]. Integrin precursor subunits are glycosylated and assembled in the ER and Golgi, from where they are transported to the plasma membrane as mature assembled heterodimers [85,86]. Unassembled excess ITGB1 subunits are retained in the ER and degraded through the ERAD pathway [86]. In normal Schwann cells, only around 20% of newly expressed PMP22 protein is glycosylated correctly and trafficked to the plasma membrane, with the remaining misfolded protein subjected to endoplasmic reticulum-associated degradation (ERAD) [4]. It has been suggested that overexpression of PMP22 causes the ER protein folding quality control system to be overwhelmed, causing the accumulation of cytotoxic aggregates, an increase in misfolding and mistrafficking of PMP22, and Schwann cell dysfunction [10,87]. As illustrated in Figure 7A, in normal Schwann cells, newly synthesised integrins and PMP22 are processed in the ER and Golgi, with mature proteins transported to the plasma membrane, where integrin dimers interact with the extracellular matrix, in the proximity of PMP22, for myelination to occur. In CMT1A Schwann cells, as illustrated in Figure 7B, there are increased levels of PMP22, ITGA2 and ITGA7. The accumulation of misfolded PMP22 in the ER leads to oversaturation of the ER quality control network and this may interfere with the normal processing and trafficking of integrins. It could be speculated that overexpression of PMP22 at the plasma membrane may alter the conformation of integrin dimers, perhaps by steric hindrance or other mechanisms of interference, which disrupts the interaction between Schwann cells and the extracellular matrix, and so lead to defective myelination in CMT1A. Our results suggest that when PMP22 is overexpressed maximum levels of ITGA2 occur at the plasma membrane at 3 d culture, whereas higher levels of ITGA7 are expressed throughout the cells, with the highest levels of expression of ITGA7 occurring as a later event, further down the myelination pathway in the 10 d-treated cultures.

## 5. Conclusions

The CMT1A cell model described here is reproducible, cost-effective and operationally uncomplicated. It has been applied to demonstrate molecular pathways and interactions, such as the interaction between overexpressed PMP22 and ITGA2 and ITGA7, which are expressed on the plasma membrane of Schwann cells and are involved in myelination via their interactions with the extracellular matrix. In recent years there have been great advances in understanding CMT and in testing potential therapies, but effective drug treatments are not yet available [88]. We suggest that the present cell model may complement existing models in understanding the pathophysiological mechanisms of CMT1A and highlighting potential targets for therapy.

## Figures and Tables

**Table 1:** Cell lines doubling times, cell counts and Ki67 quantitation (mitotic activity).

	Population-Doubling Time (Hours) ^a^	Cells/cm^2^ (3 Days) ^b^		Ki67 Quantitation (3 Days): Mean Grey Value per Nucleus ^e^ Mean ± SD (*n* = 50)
		Adherent Mean ± SD (*n* = 4)	Non-Adherent Mean ± SD (*n* = 4)	
CRL-3392	22.3	41,400 ± 4400	77 ± 14	33.4 ± 13.0
Control-transfected	23.0	34,600± 2875	16 ± 6	20.8 ± 6.0
PMP22-transfected	30.7	22,000± 6866 (*) ^c^	696 ± 108 (***) ^d^	13.6 ± 10.7 (***) ^f^

^a^ Population-doubling time during exponential growth was calculated as the time taken for cell numbers to increase from 2 × 10^4^ to 4 × 10^4^ cells/cm^2^. ^b^ The 3 cell lines were passaged at 2 × 10^4^ cells/mL (4 × 10^3^ cells/cm^2^) and cultured for 3 days. There were significantly fewer adherent PMP22-transfected cells ^c^ and significantly more non-adherent PMP22-transfected cells ^d^, compared to both control and CRL cells. ^e^ The 3 cell lines were passaged, cultured for 3 days and Ki67 quantitated. ^f^ PMP22-transfected cells had significantly less Ki67 compared to both control and CRL cells. *T*-test: * *p* < 0.05; *** *p* < 0.001.

**Table 2:** PMP22 protein expression of Schwann cells determined by quantitation of immunofluorescence staining.

	3 d	10 d
CRL-3392	0.28 ± 0.11	1.28 ± 0.60 **
Control-trans	0.05 ± 0.02	1.61 ± 0.74 ***
PMP22-trans	4.19 ± 2.79	4.85 ± 1.95

Mean grey value per cell ± SD (each value obtained from *n* = 6 to 9 fields). See Figure 1C. CRL and control-transfected cell lines had greater immunofluorescence staining at 10 days compared with the same cell line at 3 days. *T*-test: ** *p* < 0.01; *** *p* < 0.001.

**Table 3:** Mass spectrometry abridged results. Top 12 proteins found only in the PMP22 pull-down.

Protein	Accession	Number of Significant Peptides
Isoform 2 of Extended synaptotagmin-1 (ESYT1)	Q9BSJ8-2	28
Integrin alpha-2 (ITGA2)	P17301	20
Treacle protein (TCOF1)	Q13428	19
Structural maintenance of chromosomes protein (SMC1A)	G8JLG1	18
Plasma membrane calcium-transporting ATPase 1 (ATP2B1)	P20020	17
DNA replication licencing factor MCM5 (MCM5)	P33992	16
Glutamine--tRNA ligase (QARS1)	P47897	16
Isoform 13 of Sodium bicarbonate cotransporter 3 (SLC4A7)	Q9Y6M7-13	16
Phospholipid-transporting ATPase IC (ATP8B1)	O43520	15
Polyadenylate-binding protein 1 (PABPC1)	A0A7I2V649	15
Integrin alpha-7 (ITGA7)	J3KNV4	14
Vigilin (Fragment) (HDLBP)	H0Y394	14

## Data Availability

The raw data supporting the conclusions of this article will be made available by the authors on request.

## Supplementary Materials

The following supporting information can be downloaded at https://www.mdpi.com/article/10.3390/biology14111552/s1, Supplementary File S1 (.xlsx): Mass spectrometry results. Supplementary File S2 (.PDF): Figure S1, Plasmids; Figure S2, Live cell microscopy; Figure S3, Immunofluorescence staining for BioID2; Figure S4, Immunofluorescence staining for Ki67; Figure S5, Immunofluorescence staining for pan–ESYT1; Table S1, Quantitative PCR primer pairs [20,36,37]. Supplementary File S3 (.PDF): Original uncropped blots used for Figure 2, Figure 3 and Figure 6.

## Abbreviations

The following abbreviations are used in this manuscript:

BioID	Proximity-dependent biotin identification
BioID2	Proximity-dependent biotin identification 2
CRL	CRL-3392. Parent cell line. Immortalised human Schwann cell
CMT1	Charcot–Marie–Tooth disease type 1
CMT1A	Charcot–Marie–Tooth disease type 1A
CMT2	Charcot–Marie–Tooth disease type 2
DPBS	Dulbecco’s phosphate-buffered saline
EGF	Epidermal growth factor
EGFP	Enhanced green fluorescent protein
EGR2	Early growth response 2; KROX20
ER	Endoplasmic reticulum
ERAD	Endoplasmic reticulum-associated degradation
ESYT1	Extended synaptotagmin-1
hiPSC	Human induced pluripotent stem cells
HNPP	Hereditary neuropathy with pressure palsies
HOXC4	Homeobox C4
IPA	Ingenuity Pathway Analysis
ITGA2	Integrin alpha 2
ITGA7	Integrin alpha 7
ITGB1	Integrin beta 1
JUN	Jun proto-oncogene, AP-1 transcription factor subunit; c-JUN
MPZ	Myelin protein zero; P0; MPP
NGFR	Nerve growth factor receptor; P75NTR
NRG1	Neuregulin 1
PAGE	Polyacrylamide gel electrophoresis
PALS1	Protein associated with LIN7 1, MAGUK P55 family member
PBS	Phosphate-buffered saline
PMP22	Peripheral myelin protein 22
RIPA buffer	Radioimmunoprecipitation assay buffer
S100B	S100 calcium binding protein B
SDS	Sodium dodecyl sulfate
SOX10	SRY-Box transcription factor 10
