# Supplementary material for: Characterising PMP22-Proximal Partners in a Schwann Cell Model of Charcot–Marie–Tooth Disease Type1A
Source: Biology (Basel). 2025 Nov 5;14(11):1552. doi: 10.3390/biology14111552 (PMC12650596; doi:10.3390/biology14111552)
Supplement: Supplementary file 1 [file biology-14-01552-s001.zip › Holt et al Supplementary File S2.pdf]

Supplementary File 2

Characterising PMP22 proximal partners in a Schwann cell model of Charcot-Marie-Tooth disease type1A

Ian Holt, Nicholas Emery, Monte A Gates, Sharon J Brown, Sally L Shirran, and Heidi R Fuller

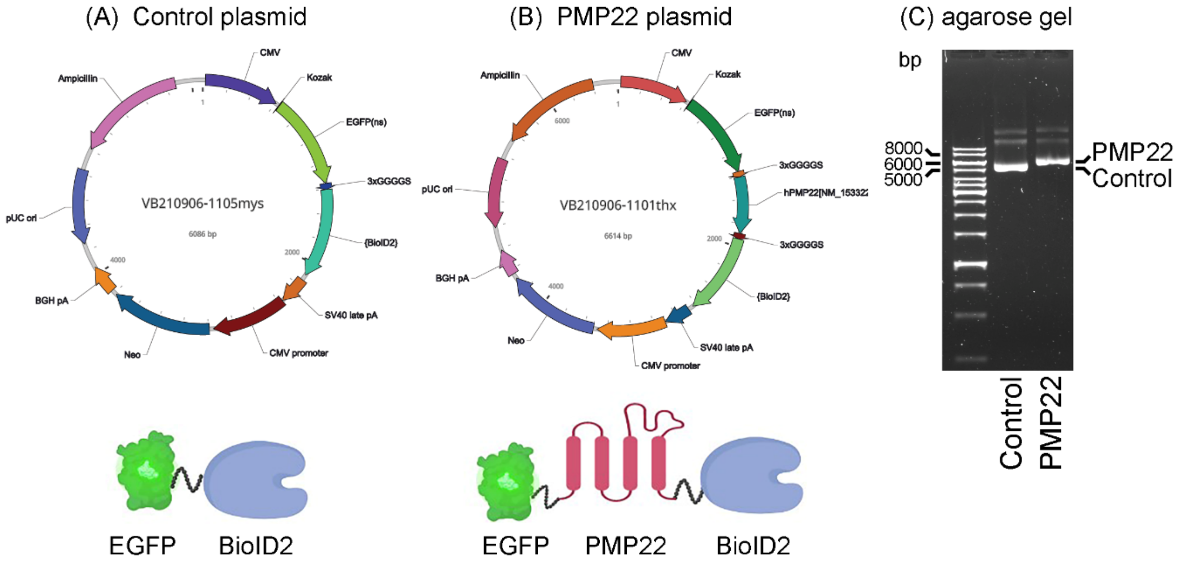

Figure S1

Plasmids: Plasmid maps showing (A) Control and (B) PMP22 vector designs, reproduced with permission from VectorBuilder.com. Below the plasmid maps are schematic representations of the two recombinant proteins that are expected to be expressed. (C) Agarose gel electrophoresis showing control and PMP22 plasmid preparations at approximately expected sizes of 6086 and 6614bp respectively.

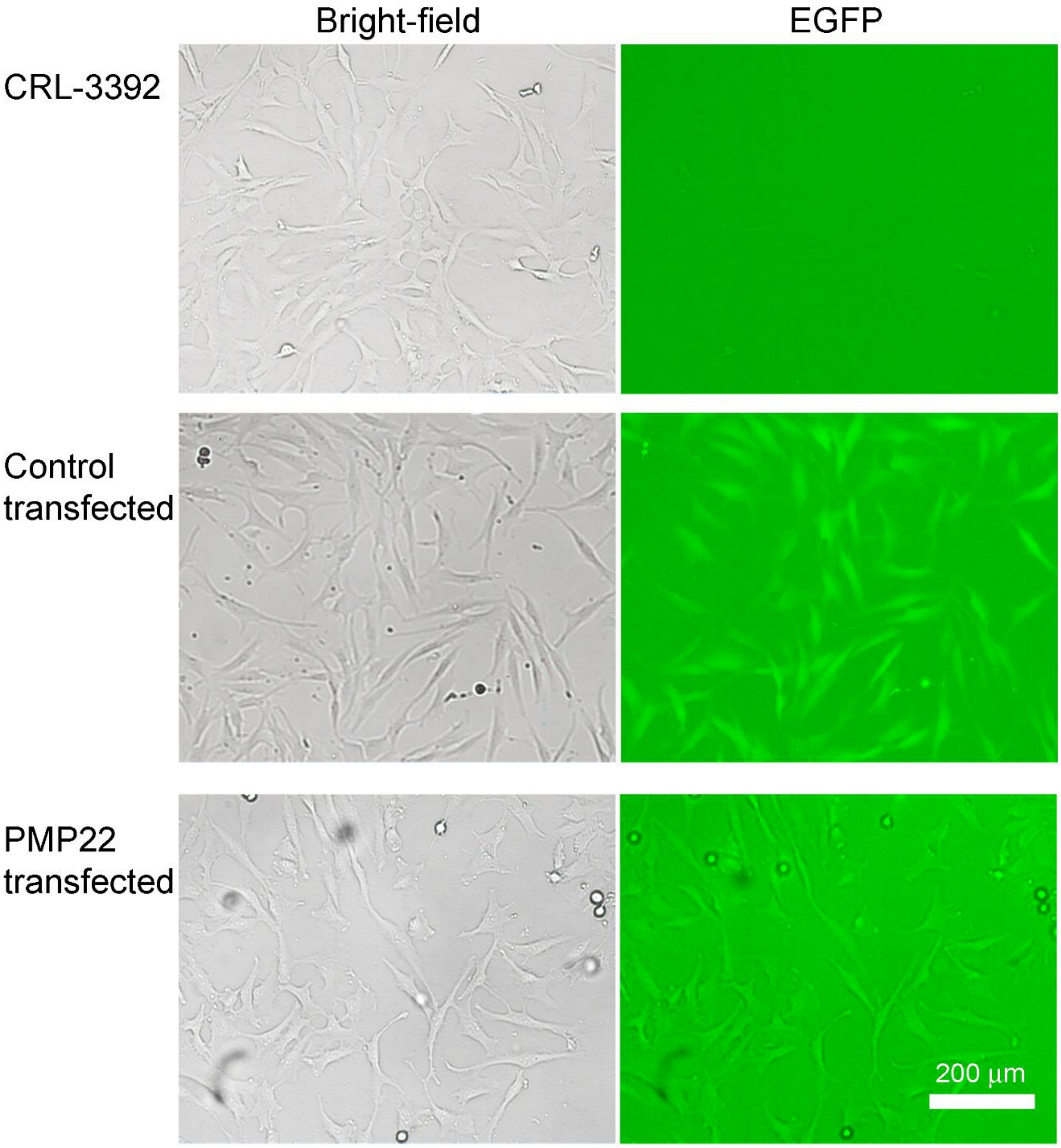

Figure S2

Live cell microscopy: Following the second cloning, all of the cells in the two transfected cell lines expressed EGFP, indicating clonality.

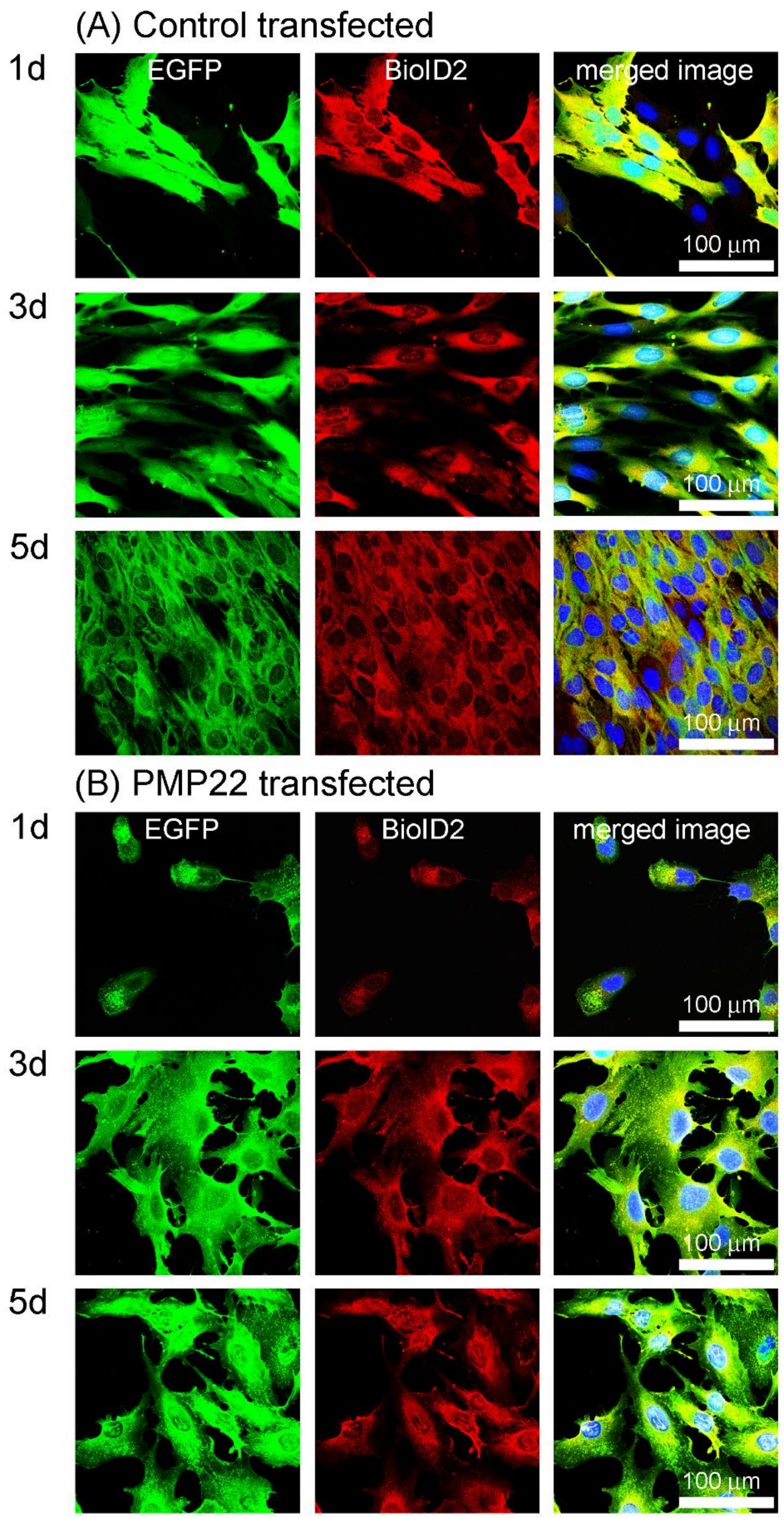

Figure S3

Immunofluorescence staining for BioID2: As a second screen, transfected cells were passaged and fixed after 1, 3 and 5 days and immuno-stained for BioID2. BioID2 colocalised with EGFP in both of the transfected cell lines, indicating clonality.

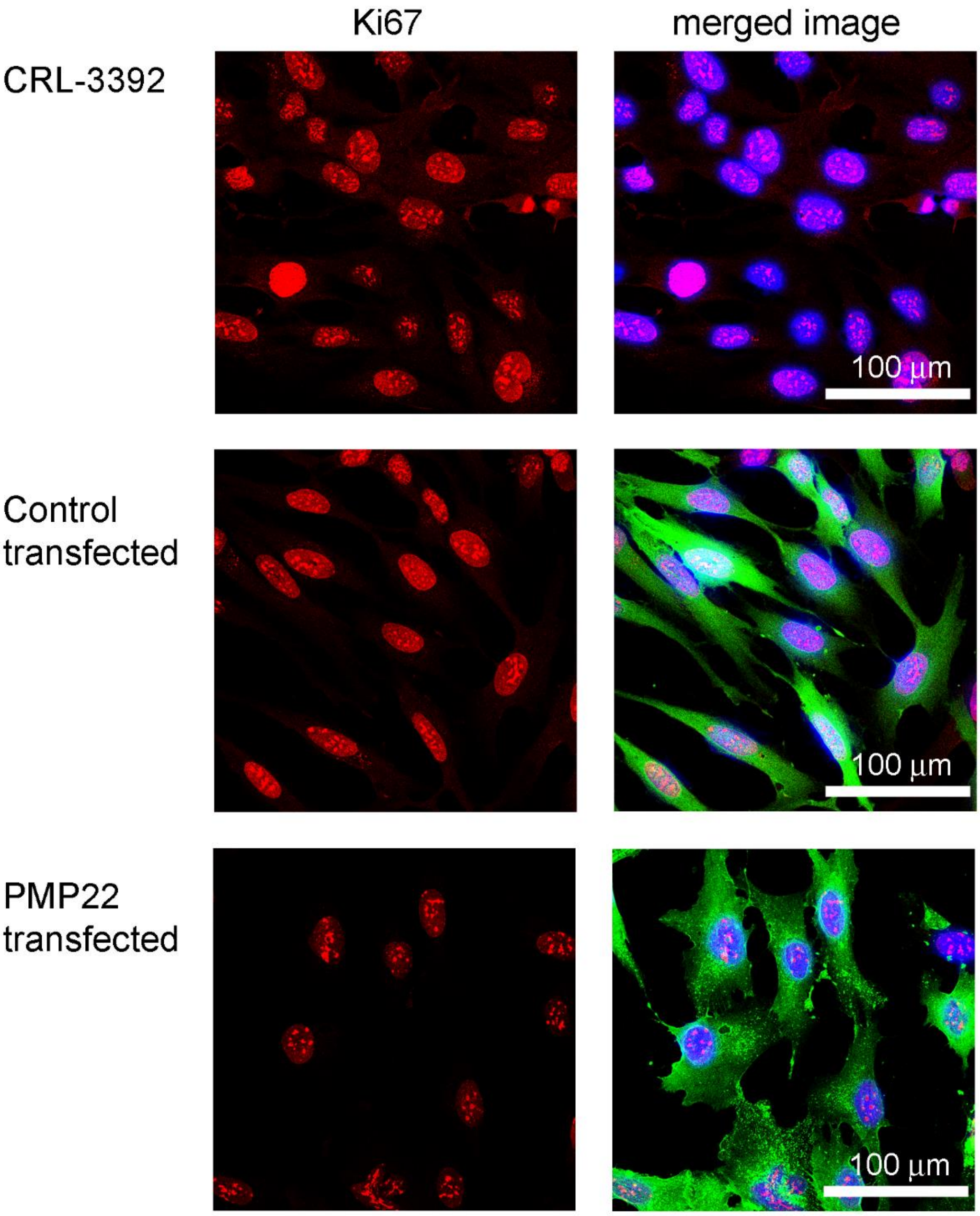

Figure S4

Immunofluorescence staining for Ki67: Ki67 locates to structures including nucleoli and is a marker of proliferation and mitosis. Quantitation of Ki67 (See Table 1) showed less Ki67 and therefore lower mitotic potential in PMP22-transfected cells compared to the other two cell lines.

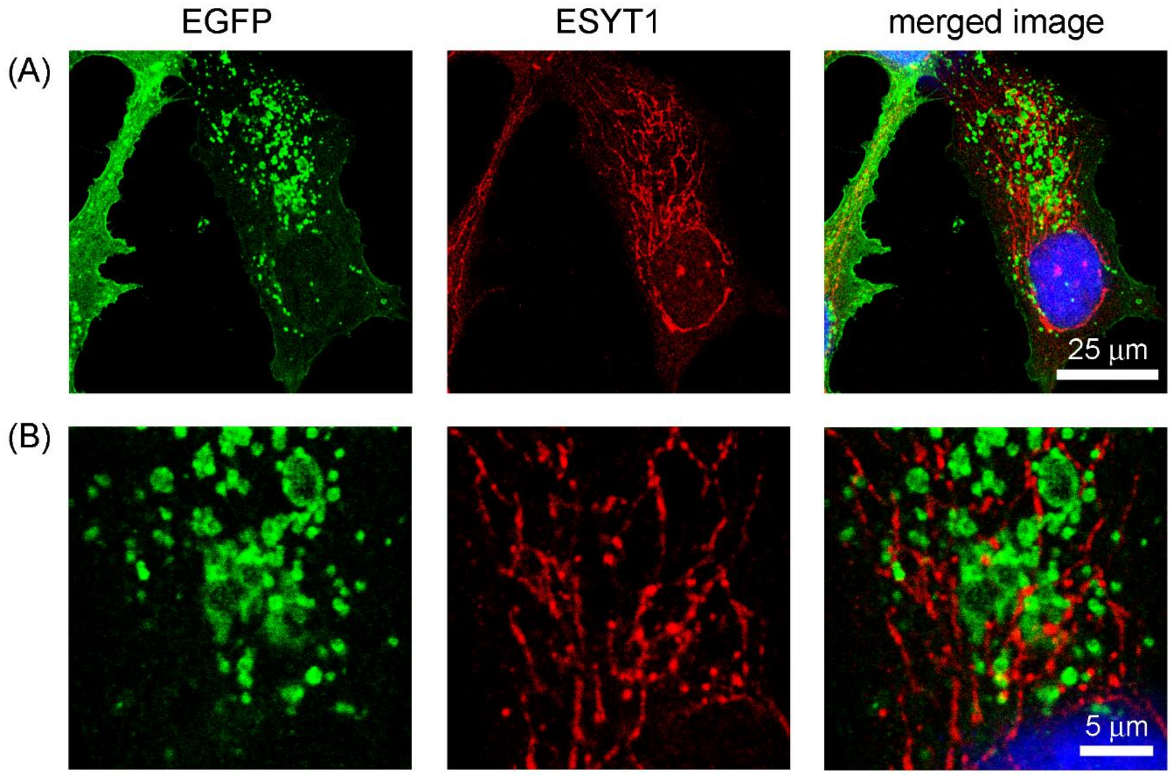

Figure S5

Immunofluorescence staining for pan - ESYT1 in PMP22-transfected cells: ESYT1 is a component of the endoplasmic reticulum membrane. Transfected PMP22-EGFP often appeared to localise within endoplasmic reticulum. The upper image (A) shows ESYT1 (red) in a cell transfected with PMP22 (green). The lower image (B) is an enlarged area of the upper image, showing more detail of the endoplasmic reticulum and other structures.

Table S1: Quantitative PCR primer pairs

Target (GenBank Accession)	Primer Sequence 5' to 3'	Product Size (bp)	Reference
Beta-actin (NM_001101)	F: CCCTGGAGAAGAGCTACGAG<br>R: TGAAGGTAGTTTCGTGGATGC	135	[36] Duong et al., 2014
GAPDH (NM_002046)	F: CAGCCTCAAGATCATCAGCA<br>R: GTCTTCTGGGTGGCAGTGAT	135	[36] Duong et al., 2014
PMP22 (NM_153322.3)	F: ACTGTAGCACCTCTTCCTCAGG<br>R: GCAGCACTCATCACGCACAGAC	220	Used here for the first time
BioID2 (MQ048429.1)	R: CTCCTTCAGCCAGATCAGGTTC<br>(Paired with PMP22 forward primer)	434<br>(F: PMP22<br>R: BioID2)	Used here for the first time
MPZ (NM_000530)	F: CATCGTGGTTTACACCGACAG<br>R: TGGAAGATCGAAATGGCATCTCT	156	[20] Shi et al., 2018
EGR2 (NM_001136179)	F: TCAACATTGACATGACTGGAGAG<br>R: AGTGAAGGTCTGGTTTCTAGGT	74	[37] Harvard PrimerBank ID: 209969756c1
SOX10 (NM_006941)	F: CCTCACAGATCGCCTACACC<br>R: CATATAGGAGAAGGCCGAGTAGA	139	[37] Harvard PrimerBank ID: 30179898c1
HOXC4 (NM_014620)	F: GCCAGCAAGCAACCCATAGT<br>R: CCTTCTCCTTCGGGTCAGGT	174	[20] Shi et al., 2018
JUN (NM_002228)	F: TCCAAGTGCCGAAAAAGGAAG<br>R: CGAGTTCTGAGCTTTCAAGGT	58	[37] Harvard PrimerBank ID: 44890066c1
S100B (NM_006272)	F: TGGCCCTCATCGACGTTTTC<br>R: ATGTTCAAAGAACTCGTGGCA	248	[20] Shi et al., 2018
NGFR (NM_002507)	F: CCTACGGCTACTACCAGGATG<br>R: CACACGGTGTTCTGCTTGT	91	[37] Harvard PrimerBank ID: 295842401c1
ITGA2 (NM_002203)	F2: GGGAATCAGTATTACACAACGGG<br>R2: CCACAACATCTATGAGGGAAGGG	90	[37] Harvard PrimerBank ID: 116295257c2
ITGA7 (NM_001144997)	F: CAGCGAGTGGACCAGATCC<br>R: CCAAAGAGGAGGTAGTGGCTATC	181	[37] Harvard PrimerBank ID: 222418614c1
ITGB1 (NM_033668)	F: CCTACTTCTGCACGATGTGATG<br>R: CCTTTGCTACGGTTGGTTACATT	106	[37] Harvard PrimerBank ID: 182507160c1
