# Supplementary material for: Characterising PMP22-Proximal Partners in a Schwann Cell Model of Charcot–Marie–Tooth Disease Type1A
Source: Biology (Basel). 2025 Nov 5;14(11):1552. doi: 10.3390/biology14111552 (PMC12650596; doi:10.3390/biology14111552)

Supplementary File 3: Original uncropped blots

Characterising PMP22 proximal partners in a Schwann cell model of Charcot-Marie-Tooth disease type1A

Ian Holt, Nicholas Emery, Monte A Gates, Sharon J Brown, Sally L Shirran, and Heidi R Fuller

Vertical black arrows show the lanes of the blots that were used in the final figures.

Original blot used for Figure 2C

Western blot for PMP22
Chemiluminescent image.

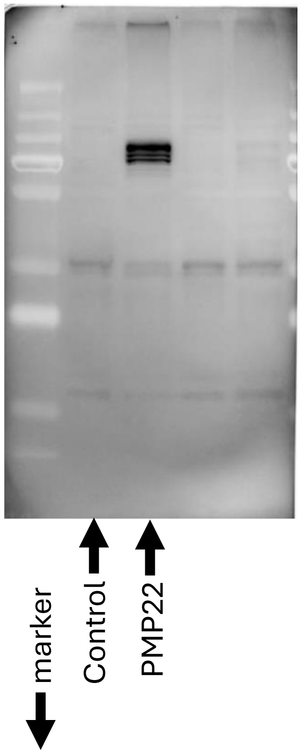

Colourimetric image, showing molecular weight markers.

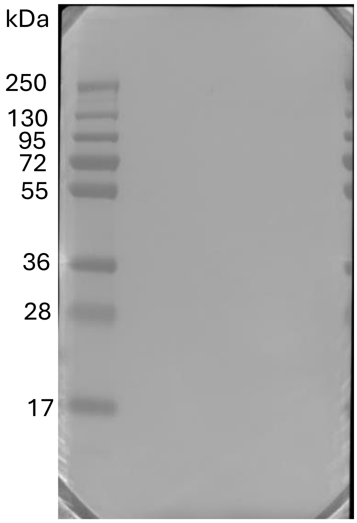

Original blot used for Figure 2D

Western blot for EGFP
Chemiluminescent image.

Colourimetric image, showing molecular weight markers.

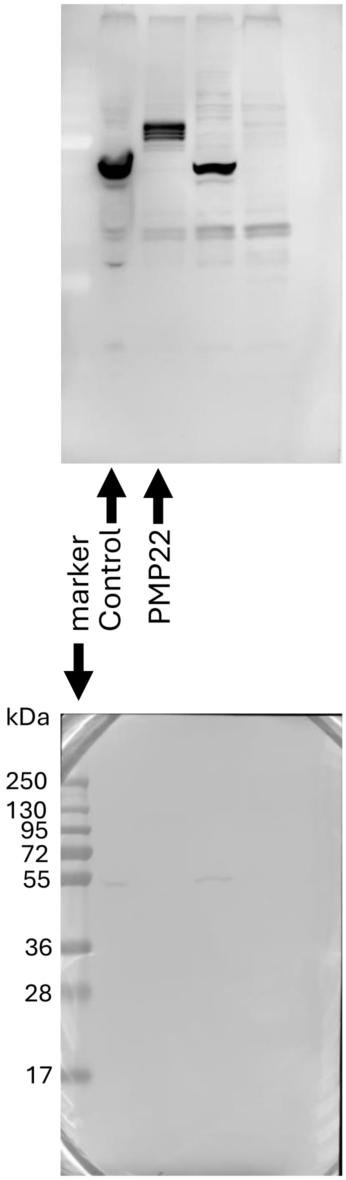

Original blot used for Figure 2E

Western blot for BioID2
Chemiluminescent image.

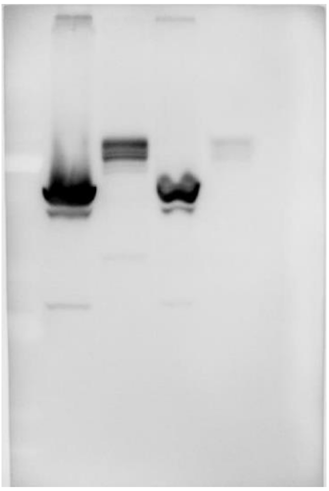

Colourimetric image, showing molecular weight markers.

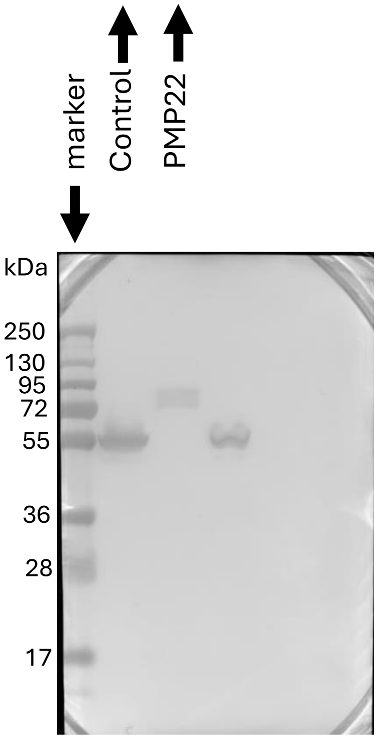

Original membrane used for Figure 3B

Nitrocellulose membrane probed for Biotin
Chemiluminescent image.
Relative densitometry measurements of the lanes are shown in red.

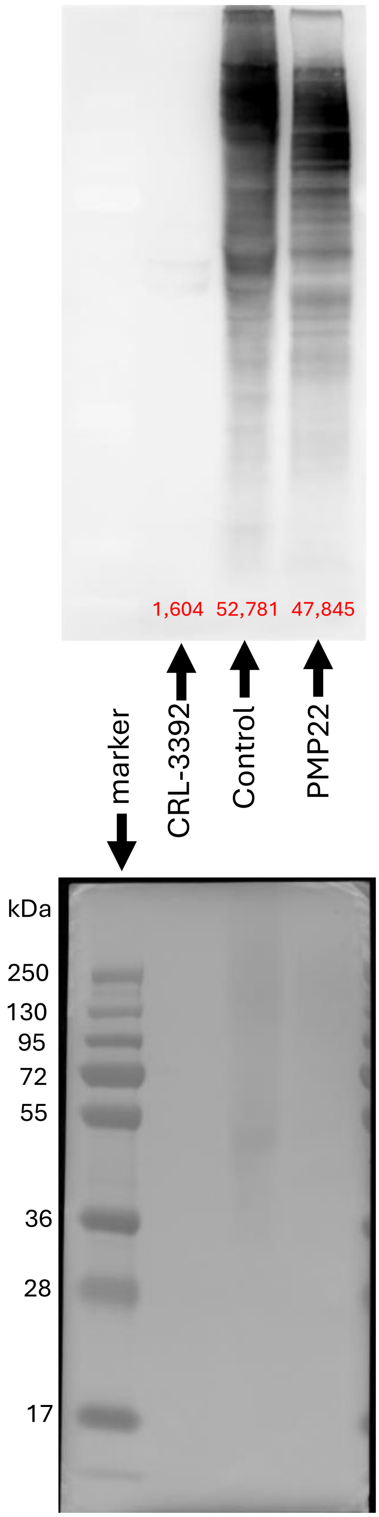

Colourimetric image, showing molecular weight markers

Original blot used for Figure 6A

Western blot for ITGA2
Chemiluminescent image of nitrocellulose membrane, transferred from the upper part of the gel. Relative densitometry measurements of the ITGA2 bands are shown in red.

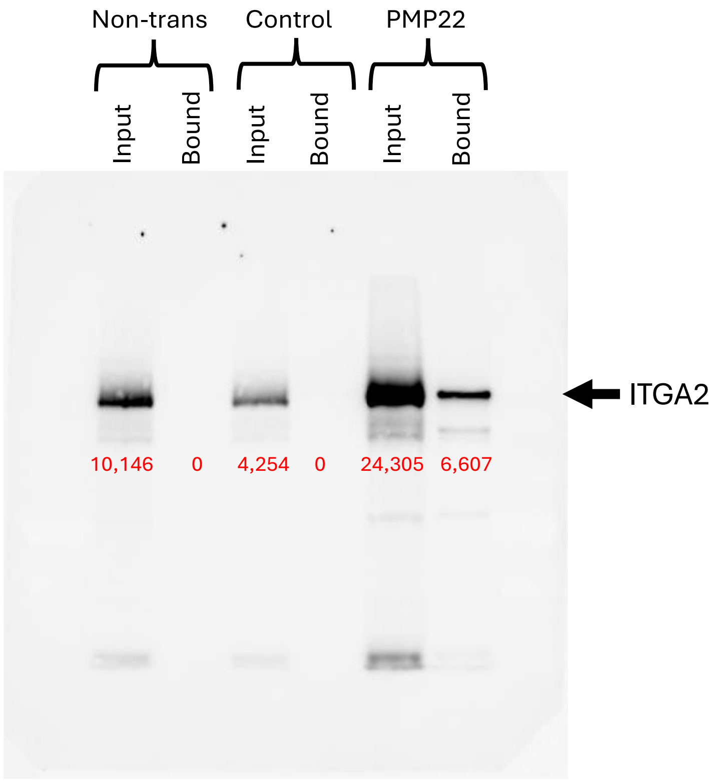

Colourimetric image of nitrocellulose membrane, transferred from the upper part of the gel, showing molecular weight markers 50 to 185 kDa.

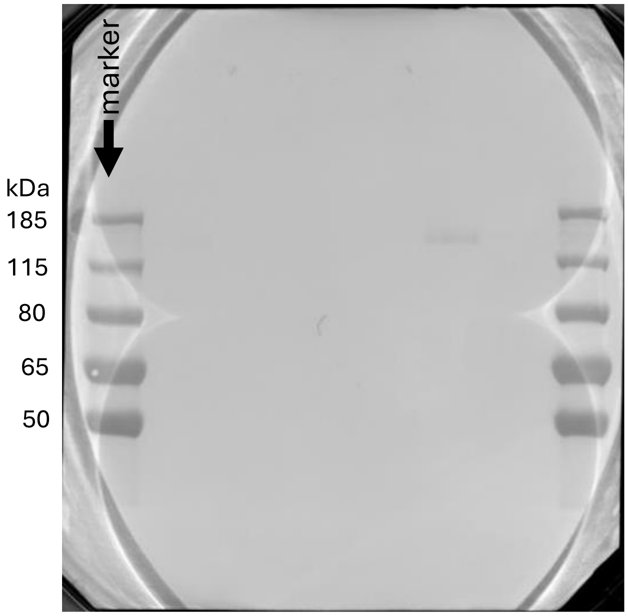

Lower part of gel, stained with Coomassie blue, showing molecular weight markers 10 to 30 kDa and protein staining of input and bound samples, between these molecular weights. Relative densitometry measurements of the lanes are shown in red.

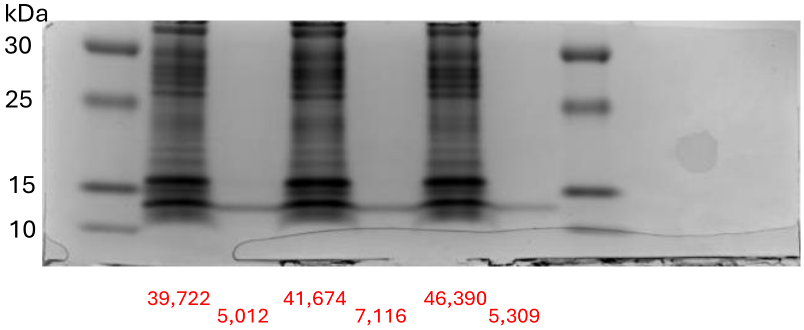

Supplement: Supplementary file 1 [file biology-14-01552-s001.zip › Holt et al Supplementary File S3 - original blots.pdf]
